# IMPI: Making MPI Interoperable

**DOI:** 10.6028/jres.105.035

**Published:** 2000-06-01

**Authors:** William L. George, John G. Hagedorn, Judith E. Devaney

**Affiliations:** National Institute of Standards and Technology, Gaithersburg, MD 20899-8591

**Keywords:** conformance testing, distributed processing, interoperable, message passing, MPI, parallel processing

## Abstract

The Message Passing Interface (MPI) is the de facto standard for writing parallel scientific applications in the message passing programming paradigm. Implementations of MPI were not designed to interoperate, thereby limiting the environments in which parallel jobs could be run. We briefly describe a set of protocols, designed by a steering committee of current implementors of MPI, that enable two or more implementations of MPI to interoperate within a single application. Specifically, we introduce the set of protocols collectively called Interoperable MPI (IMPI). These protocols make use of novel techniques to handle difficult requirements such as maintaining interoperability among all IMPI implementations while also allowing for the independent evolution of the collective communication algorithms used in IMPI. Our contribution to this effort has been as a facilitator for meetings, editor of the IMPI Specification document, and as an early testbed for implementations of IMPI. This testbed is in the form of an IMPI conformance tester, a system that can verify the correct operation of an IMPI-enabled version of MPI.

## 1. Introduction

The Message Passing Interface (MPI) [6,7] is the de facto standard for writing scientific applications in the message passing programming paradigm. MPI was first defined in 1993 by the MPI Forum (http://www.mpi-forum.org), comprised of representatives from United States and international industry, academia, and government laboratories. The protocol introduced here, the Interoperable MPI protocol (IMPI),^1^ extends the power of MPI by allowing applications to run on heterogeneous clusters of machines with various architectures and operation systems, each of which in turn can be a parallel machine, while allowing the program to use a different implementation of MPI on each machine. This is accomplished without requiring any modifications to the existing MPI specification. That is, IMPI does not add, remove, or modify the semantics of any of the existing MPI routines. All current valid MPI programs can be run in this way without any changes to their source code.

The purpose of this paper is to introduce IMPI, indicate some of the novel techniques used to make IMPI work as intended, and describe the role NIST has played in its development and testing. As of this writing, there is one MPI implementation, Local Area Multicomputer (LAM) [19], that supports IMPI, but others have indicated their intent to implement IMPI once the first version of the protocol has been completed. A more detailed explanation of the motivation for and design of IMPI is given in the first chapter of the IMPI Specification document [3], which is included in its entirety as an appendix to this paper.

The need for interoperable MPI is driven by the desire to make use of more than one machine to run applications, either to lower the computation time or to enable the solution of problems that are too large for any available single machine. Another anticipated use for IMPI is for computational steering in which one or more processes, possibly running on a machine designed for high-speed visualization, are used interactively to control the raw computation that is occurring on one or more other machines.

Although current portable implementations of MPI, such as MPICH [14] (from the MPICH documentation: *The “CH” in MPICH stands for “Chameleon,” symbol of adaptability to one’s environment and thus of portability.*), and LAM (Local Area Multicomputer) [2] support heterogeneous clusters of machines, this approach does not allow the use of vendor-tuned MPI libraries and can therefore sacrifice communications performance. There are several other related projects. PVMPI [4] (PVMPI is a combination of the acronyms PVM, which stands for Portable Virtual Machine (another message passing system) and MPI) and its successor MPI-Connect [5], use the native MPI implementation on each system, but use some other communication channel, such as PVM, when passing messages between processes in the different systems. One main difference between the PVMPI/MPI-Connect interoperable MPI systems and IMPI is that no collective communication operations, such as broadcasting a value from one process to all of the other processes (MPI_Bcast) or synchronizing all of the processes (MPI_Barrier), are supported between MPI implementations. MAGPIE [16,17] is a library of collective communications operations built on top of MPI (using MPICH) and optimized for wide area networks. Although this system allows for collective communications across all MPI processes, you must use MPICH on all of the machines and not the vendor tuned MPI libraries. Finally, MPICH-G [8], is a version of MPICH developed in conjunction with the Globus project [9] to operate over a wide area network. This also bypasses the vendor tuned MPI libraries.

Several ongoing research projects take the concept of running parallel applications on multiple machines much further. The concept variously known as *meta-computing*, *wide area computing*, *computational grids*, or the IPG (Information Power Grid), is being pursued as a viable computational framework in which a program is submitted to run on a geographically distributed group of Internet-connected sites. These sites form a *grid* which provides all of the resources, including multiprocessor machines, needed to run large jobs. The many and varied protocols and infrastructures needed to realize this is an active research topic [9,10,11,12, 13,15]. Some of the problems under study include computational models, resource allocation, user authentication, resource reservations, and security. A related project at NIST is WebSubmit [18], a web-based user interface that handles user authentication and provides a single point of contact for users to submit and manage long running jobs on any of our high-performance and parallel machines.

## 2. The IMPI Steering Committee Meetings

The Interoperable MPI steering committee first met in March 1997 to begin work on specifying the Interoperable MPI protocol. This first meeting was organized and hosted by NIST at the request of the attending vendor and academic representatives. All of these initial members (with one neutral exception) expressed the view that the role of NIST in this process would be vital. As a knowledgeable neutral party, NIST would help facilitate the process and provide a testbed for implementations. At this first meeting, only representatives from within the United States attended, but the question of allowing international vendors to participate was introduced. This was later agreed to and several foreign vendors actively participated in the IMPI meetings. All participating vendors are listed in the IMPI document (see Appendix).

There were eight formal meetings of the IMPI steering committee from March 1997 to March 1999, augmented with a NIST-maintained mailing list for ongoing discussions between meetings.

NIST has had three main roles in this effort: facilitator for meetings and maintaining an on-line mailing list, editor for the IMPI protocol document, and conformance testing. It is this last task, conformance testing, that required our greatest effort.

## 3. Design Highlights of the IMPI Protocols

The IMPI protocols were designed with several important guiding principles. First, IMPI was not to alter the MPI interface. That is, no user level MPI routines were to be added and no changes were to be made to the interfaces of the existing routines. Any valid MPI program must run correctly using IMPI if it runs correctly without IMPI. Second, the performance of communication within an MPI implementation should not be noticeably impacted by supporting IMPI. IMPI should only have a noticeable impact on communication performance when a message is passed between two MPI implementations (the success of this goal will not be known until implementations are completed). Finally, IMPI was designed to allow for the easy evolution of its protocols, especially its collective communications algorithms. It is this last goal that is most important for the long-term usefulness of IMPI for MPI users.

An *IMPI job*, once running, consists of a set of MPI processes that are running under the control of two or more instances of MPI libraries. These MPI processes are typically running on two or more *systems*. A system, for this discussion, is a machine, with one or more processors, that supports MPI programs running under control of a single instance of an MPI library. Note that under these definitions, it is not necessary to have two different implementations of MPI in order to make good use of IMPI. In fact, given two identical multiprocessor machines that are only linked via a LAN (Local Area Network), it is possible that the vendor supplied MPI library will not allow you to run a single MPI job across all of the processors of both machines. In this case, IMPI would add that capability, even though you are running on one architecture and using one implementation of MPI.

The remainder of this section outlines some of the more important design decisions made in the development of IMPI. This is a high-level discussion of a few important aspects of IMPI with many details omitted for brevity.

### 3.1 Common Communication Protocol

As few assumptions as possible were made about the systems on which IMPI jobs would be run; however some common attributes were assumed in order to begin to obtain interoperability.

The most basic assumption made, after some debate, was that TCP/IP would be the underlying communications protocol between IMPI implementations. TCP/IP (Transmission Control Protocol/Internet Protocol), is one of the basic communications protocols used over the Internet. It is important to note that this decision does not mandate that all machines running the MPI processes be capable of communicating over a TCP/IP channel, only that they can communicate, directly or indirectly, with a machine that can. IMPI does not require a completely connected set of MPI processes. In fact, only a small number of communications channels are used to connect the MPI processes on the participating systems.

The decision to use only a few communications channels to connect the systems in an IMPI job, rather than requiring a more dense connection topology, was made under the assumption that these IMPI communications channels would be slower, in some cases many times slower, than the networks connecting the processors within each of the systems. Even as the performance of networking technology increases, it is likely that the speed of the dedicated internal system networks will always meet or exceed the external network speed.

Other communications mediums, besides TCP/IP, could be added to IMPI as needed, for example to support IMPI between embedded devices. However, the use of TCP/IP was considered the natural choice for most computing sites.

### 3.2 Start-up

One of the first challenges faced in the design of IMPI was determining how to start an IMPI job. The main task of the IMPI start-up protocol is to establish communication channels between the MPI processes running on the different systems.

Initially, several procedures for starting an IMPI job were proposed. After several iterations a very simple and flexible system was designed. A single, implementation-independent process, the *IMPI server*, is used as a rendezvous point for all participating systems. This process can be run anywhere that is network-reachable by all of the participating systems, which includes any of the participating systems or any other suitable machine. Since this server utilizes no architecture specific information, a portable implementation can be shared by all users. As a service to the other MPI implementors, the Laboratory for Computer Science at the University of Notre Dame (the current developers of LAM/MPI), has provided a portable IMPI server that all vendors can use. The IMPI server is not only implementation independent, it is also immune to most changes to IMPI itself. The server is a simple rendezvous point that knows nothing of the information it is receiving; it simply relays the information it receives to all of the participating systems. All of the negotiations that take place during the start-up are handled within the individual IMPI/MPI implementations. The only information that the server needs at start-up is how many systems will be participating.

One of the first things the IMPI server does is print out a string containing enough information for any of the participating systems to be able to contact it. This string contains the Internet address of the machine running the IMPI server and the TCP/IP port that the server is listening on for connections from the participating systems.

The conversation that takes place between the participating systems, relayed through the IMPI server, is in a simple “tokenized” language in which each token identifies a certain piece of information needed to configure the connections between the systems. For example, one particular token exchanged between all systems indicates the maximum number of bytes each system is willing to send or receive in a single message over the IMPI channels. Messages larger that this size must be divided into multiple packets, each of which is no larger than this maximum size. Once this token is exchanged, all systems choose the smallest of the values as the maximum message size.

Many tokens are specified in the IMPI protocol, and all systems must submit values for each of these tokens. However, any system is free to introduce new tokens at any time. Systems unfamiliar with any token it receives during start-up can simply ignore it. This is a powerful capability that requires no changes to either the IMPI server, or to the current IMPI specification. This allows for experimentation with IMPI without requiring the active participation of other IMPI/MPI implementors. Once support for IMPI version 0.0 has been added to them, any of the freely available implementations of MPI, such as MPICH or LAM, can be used by anyone interested in experimenting with IMPI at this level. If a new start-up parameter appears to be useful, then it can be added to an IMPI implementation and be used as if it were part of the original IMPI protocol.

One particular parameter, the IMPI version number, is intended for indicating updates to one or more internal protocols or to indicate the support for a new set of collective communications algorithms. For example, if one or more new collective algorithms have been shown to enhance the performance of IMPI, then support for those new algorithms by a system would be indicated by passing in the appropriate IMPI version number during IMPI start-up. All systems must support IMPI version 0.0 level protocols and collective communications algorithms, but may also support any number of higher level sets of algorithms. This is somewhat different than traditional version numbering in that an IMPI implementation must indicate not only its latest version, but all of the previous versions that it currently supports (which must always include 0.0). Since all systems must agree on the collective algorithms to be used, the IMPI version numbers are compared at start-up and the highest version supported by all systems will be used. It is possible for an IMPI implementation to allow the user to control this negotiation partially by allowing the user to specify a particular IMPI version number (as a command-line option perhaps). The decision to provide this level of flexibility to the user is completely up to those implementing IMPI.

### 3.3 Security

As an integral part of the IMPI start-up protocol, the IMPI server accepts connections from the participating systems. In the time interval between the starting of the IMPI server and the connection of the last participating system to the server, there is the possibility that some other rogue process might try to contact the server. Therefore, it is important for the IMPI server to authenticate the connections it accepts. This is especially true when connecting systems that are either geographically distant or not protected by other security means such as a network firewall. The initial IMPI protocol allows for authentication via a simple 64 bit key chosen by the user at start-up time. Much more sophisticated authentication systems are anticipated so IMPI includes a flexible security system that supports multiple authentication protocols in a manner similar to the support for multiple IMPI versions. Each IMPI implementation must support at least the simple 64 bit key authentication, but can also support any number of other authentication schemes.

Just as the collective communications algorithms that are to be used can be partially controlled by the user via command-line options, the authentication protocol can also be chosen by the user. More details of this are given in Sec. 2.3.3 of the IMPI Specification document.

If security on the IMPI communication channels during program execution is needed, that is, between MPI processes, then updating IMPI to operate over secure sockets could be considered. Support for this option in an IMPI implementation could be indicated during IMPI start-up.

### 3.4 Topology Discovery

The topology of the network connecting the IMPI systems, that is, the set of network connections available between the systems, can have a dramatic effect on the performance of the collective communications algorithms used. It is not likely that any static collective algorithm will be optimal in all cases. Rather, these collective algorithms will need to dynamically choose an algorithm to use based on the available network. The initial IMPI collective algorithms acknowledge this in that, in many cases, they choose between two algorithms based on the size of the messages involved and the number of systems involved. Algorithms for large messages try to minimize the amount of data transmitted (do not transmit data more than once if possible) and algorithms for small messages try to minimize the latency by parallelizing the communication if possible (by using a binary tree network for a gather operation for example). In order to assist in the implementation of dynamically tunable collective algorithms, IMPI has included four topology parameters, to be made available at the user level (for those familiar with MPI, these parameters are made available as cached attributes on each MPI communicator). These attributes identify which processes are close, that is, within the same system, and which are distant, or outside the local system. Communication within a system will almost always be faster than communications between systems since communication between systems will take place over the IMPI channels. These topology attributes give no specific communications performance information, but are provided to assist in the development of more dynamic communications algorithms.

Through NIST’s SBIR (Small Business Innovative Research) program, we have solicited help in improving collective communications algorithms for IMPI as well as for clustered computing in general.

## 4. Conformance Tester

The design of the IMPI tester, which we will refer to simply as the tester, is unique in that it is accessed over the Web and operates completely over the Internet. This design for a tester has many advantages over the conventional practice of providing conformance testing in the form of one or more portable programs delivered to the implementors site and compiled and run on their system. For example, the majority of the IMPI tester code runs exclusively on a host machine at NIST, regardless of who is using the tester, thus eliminating the need to port this code to multiple platforms, the need for documents instructing the users how to install and compile the system, and the need to inform users of updates to the tester (since NIST maintains the only instance of this part of the tester). There are two components of the tester that run at the user’s site. The first of these components is a small Java applet that is down-loaded on demand each time the tester is used, so this part of the tester is always up to date. Since it is written in Java and runs in a JVM (Java Virtual Machine), there is no need to port this code either. The other part of the tester that runs at the user’s site is a test interpreter (a C/MPI program) that exercises the MPI implementation to be tested. This program is compiled and linked to the vendor’s IMPI/MPI library. Since this C/MPI program is a test interpreter and not a collection of tests, it will not be frequently updated. This means that it will most likely need to be downloaded only once by a user. All updates, corrections, and additions to the conformance test suite will take place only at NIST.

This design was inspired by the work of Brady and St. Pierre at NIST and their use of Java and CORBA in their conformance testing system [1]. In their system, CORBA was used as the communication interface between the tests and the objects under test (objects defined in IDL). In our IMPI tester, since we are testing a TCP/IP-based communications protocol, we used the Java networking packages for all communications.

## 5. Enhancements to IMPI

This initial release of the IMPI protocol will enable users to spread their computations over multiple machines while still using highly-tuned native MPI implementations. This is a needed enhancement to MPI and will be useful in many settings, such as within the computing facilities at NIST. However, several enhancements to this initial version of IMPI are envisioned.

First, the IMPI collective communications algorithms will benefit from the ongoing Grid/IPG research on efficient collective algorithms for clusters and WANs [12,16,17,20]. IMPI has been designed to allow for experimenting with improved algorithms by allowing the participating MPI implementations to negotiate, at program start-up, which version of collective communications algorithms will be used. Second, although IMPI is currently defined to operate over TCP/IP sockets, a more secure version could be defined to operate over a secure channel such as SSL (Secure Socket Layer). Third, start-up of an IMPI job currently requires that multiple steps be taken by the user. This start-up process could be automated, possibly using something like Web-Submit [18], in order to simplify the starting and stopping of IMPI jobs.

IMPI-enabled clusters could be used in a WAN (Wide Area Network) environment using Globus [9], for example, for resource management, user authentication, and other management tasks needed when operating over large distances and between separately managed computing facilities. If two or more locally managed clusters can be used via IMPI to run a single job, then these resources could be described as a single resource in a grid computation so that it can be offered and reserved as a unit in the grid.

## Supplementary Material



## Footnotes

1 Certain commercial equipment, instruments, or materials are identified in this paper to foster understanding. Such identification does not imply recommendation or endorsement by the National Institute of Standards and Technology, nor does it imply that the materials or equipment identified are necessarily the best available for the purpose.
