# Supplementary material for: IMPI: Making MPI Interoperable
Source: J Res Natl Inst Stand Technol. 2000 Jun 1;105(3):343–8. doi: 10.6028/jres.105.035 (PMC4874770; doi:10.6028/jres.105.035)
Supplement: Supplementary file 1 [file j53geo_supp.pdf]

Appendix

IMPI:
Interoperable Message-Passing Interface

IMPI Steering Committee

January 2000

Protocol Version 0.0

Abstract

This document describes the industrial led effort to create a standard for an Interoperable Message-Passing-Interface (MPI). The first steering committee meeting was held on March 4, 1997.

Contents

Acknowledgments **354**

1 Introduction to IMPI **355**
1.1 Overview . . . 355
1.2 Conventions . . . 356
1.2.1 Protocol Types . . . 356
1.2.2 Data Format . . . 357
1.2.3 Host Identifiers . . . 357
1.3 Organization of this Document . . . 357

2 Startup/Shutdown **359**
2.1 Introduction . . . 359
2.2 User Steps . . . 359
2.2.1 Launching A Server . . . 359
2.2.2 Launching Clients . . . 360
2.2.3 Examples . . . 360
2.2.4 Security . . . 361
2.3 Startup Wire Protocols . . . 361
2.3.1 Introduction . . . 361
2.3.2 The IMPI Server . . . 362
2.3.3 The `AUTH` command . . . 362
2.3.4 The `IMPI` command . . . 366
2.3.5 The `COLL` command . . . 367
2.3.6 The `DONE` command . . . 374
2.3.7 The `FINI` command . . . 374
2.3.8 Shall We Dance? . . . 375
2.4 Shutdown Wire Protocols . . . 375
2.5 Client and Host Attributes . . . 376

3 Data Transfer Protocol **377**
3.1 Introduction . . . 377
3.2 Process Identifier . . . 377
3.3 Context Identifier . . . 378
3.4 Message Tag . . . 378
3.5 Message Packets . . . 378
3.6 Packet Protocol . . . 380
3.7 Message Protocols . . . 381

3.7.1 Short-Message Protocol 381
3.7.2 Long-Message Protocol 381
3.7.3 Message-Probing Protocol 382
3.7.4 Message-Cancellation Protocol 382
3.8 Finalization Protocol 383
3.9 Mandated Properties 383

4 Collectives **385**
4.1 Introduction 385
4.2 Utility functions 386
4.3 Context Identifiers 387
4.3.1 Context ID Creation 387
4.4 Comm_create 388
4.5 Comm_free 388
4.6 Comm_dup 389
4.7 Comm_split 390
4.8 Intercomm_create 391
4.9 Intercomm_merge 394
4.10 Barrier 396
4.11 Bcast 397
4.12 Gather 398
4.13 Gatherv 401
4.14 Scatter 405
4.15 Scatterv 406
4.16 Reduce 409
4.17 Reduce_scatter 412
4.18 Scan 412
4.19 Allgather 413
4.20 Allgatherv 413
4.21 Allreduce 414
4.22 Alltoall 414
4.23 Alltoallv 416
4.24 Finalize 417
4.25 Constants 417
4.26 Future work 418

5 IMPI Conformance Testing **419**
5.1 Summary 419
5.2 Test Tool Applet 424
5.3 Test Interpreter 425
5.4 Test Manager 428

Bibliography **428**

List of Figures

2.1 IMPI command exchange. 367
2.2 COLL command IMPI_C_NHOSTS exchange. 372
2.3 COLL command IMPI_H_PORT exchange. 373

5.1 IMPI Test Architecture 421
5.2 IMPI Home Page 422
5.3 IMPI Test Communications Stack 423
5.4 The Test Tool 424

List of Tables

2.1 List of standardized authentication methods, shown in enumerated and bit mask forms. 364

3.1 Packet field usage. 380

Acknowledgments

This document represents the work of the IMPI Steering Committee. The technical development of individual parts was facilitated by subgroups, whose work was reviewed by the full committee. Those who served as the primary coordinators are:

- Dean Collins: Facilitator
- Judith Devaney: Editor
- Mark Fallon and Marc Snir: Introduction to IMPI
- Eric Salo, Raja Daoud, and Jeff Squyres: Startup/Shutdown
- Raja Daoud: Data Transfer Protocol
- Nick Nevin: Collectives
- William George, John Hagedorn, Peter Ketcham, and Judith Devaney: IMPI Conformance Testing

The IMPI Steering Committee consists of the following members.

Darwin Ammala	Bill Gropp	Al Mink
Ed Benson	John Hagedorn	Jose L. Munoz
Peter Brennan	Christopher Hayden	Todd Needham
Ken Cameron	Rolf Hempel	Nick Nevin
Dean Collins	Shane Herbert	Heidi Poxon
Raja Daoud	Hans-Christian Hoppe	Nobutoshi Sagawa
Judith Devaney	Peter Ketcham	Eric Salo
Terry Dontje	Lloyd Lewins	Adam Seligman
Nathan Doss	Andrew Lumsdaine	Anthony Skjellum
Mark Fallon	Rusty Lusk	Marc Snir
Sam Feinberg	Gordon Lyon	Jeff Squyres
Al Geist	Kinis L. Meyer	Clayborne Taylor, Jr.
William George	Thom McMahon	Dick Treumann

The following institutions supported the IMPI effort through time and travel support for the people listed above.

Argonne National Laboratory
Asian Technology Information Program (ATIP)
Compaq
Defense Advanced Research Projects Agency
Digital Equipment Corp.
Fujitsu
Hewlett-Packard Co.
Hitachi
Hughes Aircraft Co.
International Business Machines
Microsoft Corp.
MPI Software Technology, Inc.
National Institute of Standards and Technology (NIST)
NEC Corp.
Oak Ridge National Laboratory
Ohio Supercomputer Center
PALLAS GmbH
Sanders, A Lockheed-Martin Co.
Silicon Graphics, Inc,
SUN Microsystems Computer Corp.
University of Notre Dame

Chapter 1

Introduction to IMPI

1.1 Overview

There is a long experience in the message passing community of harnessing heterogeneous computing resources into one parallel message passing computation. This is useful for a variety of applications: some "embarrassingly parallel" applications may be able to utilize spare compute power in a large network of workstations; some applications may decompose naturally into components that are better suited to different platforms, e.g., a simulation component and a visualization component; other applications may be too large to fit in one system.

Such applications can be developed using standard interprocess communication protocols, such as sockets on TCP/IP. However, these protocols are at a lower level than the message passing interfaces defined by MPI [1]. Furthermore, if each subsystem is a parallel system, then MPI is likely to be used for "intra-system" communication, in order to achieve the better performance that vendor MPI libraries provide, as compared to TCP/IP. It is then convenient to use MPI for "inter-system" communication as well.

MPI was designed with such heterogeneous applications in mind. For example, all message passing communication is typed, so that it is possible to perform data conversion when data is transferred across systems with different data representations. Indeed, there are several freely available implementations of MPI that run in a heterogeneous environment. These implementations use a common approach. An infrastructure is developed that provides a parallel virtual machine, on top of the multiple heterogeneous systems. Then, message passing is implemented on this parallel virtual machine. This approach has several deficiencies:

- The parallel virtual machine has to be implemented and supported on each underlying platform by a third party software developer. This poses a significant development and testing problem for such a developer, especially if it attempts to use faster but nonstandard interfaces for intra-system communication. So far, only academic development groups that have direct access to multiple platforms in supercomputing centers have been able to undertake such a development – it is hard to see a successful business model for such a product. In any case, this model implies that support for heterogeneous MPI always lags platform availability.

- Even though each system is likely to provide a native implementation of MPI for intra-system communication, the parallel virtual machine imposes an additional software layer, often resulting in reduced performance, even for MPI intra-system communication.

- The MPI standard does not specify the interaction between MPI communication and TCP/IP communication; more generally, it does not specify the interaction between the MPI imple-

mentation and the underlying system. The details are different from vendor to vendor, and from release to release by the same vendor. The details of this interaction are important for a heterogeneous MPI implementation, where a process may participate both in intra-system communication, possibly layered on top of the native MPI implementation, and in inter-system communication, possibly layered on top of sockets. The third party implementor has to "reverse engineer" the details of the vendor MPI library, and may have to make significant changes in its implementation whenever a vendor releases a new MPI implementation.

- The virtual machine library may suffer from significant inefficiencies because the internal communication layer was not built to interface with an external communication layer.

The MPI interoperability effort proposes to define a cross implementation protocol for MPI that will enable heterogeneous computing. MPI implementations that support this protocol will be able to interoperate. A parallel message passing computation will be able to span multiple systems using the native vendor message passing library on each system. We propose to do this without adding any new functions to MPI. Instead, we propose to specify implementation specific interfaces, so as to enable interoperability. In a first phase, our goal is to support all point-to-point communication functions for communication across systems, as well as collectives. We intend to phase in full MPI support, over time. The initial binding will assume that inter-system communication uses one or more sockets between each pair of communicating systems, while intra-system communication uses proprietary protocols, at the discretion of each vendor. Over time, we expect that the socket interface be expanded to allow for other industry standard stream oriented protocols, such as ATM virtual channels.

While efficient inter-system communication is important, the main performance goal of the design will be to not slow down intra-system communication: native communication performance should not be affected by the hooks added to support interoperability, as long as there is no inter-system communication. The design should be so that support for interoperability does not weaken availability and security on each system.

1.2 Conventions

In order to support heterogeneous networks a standard data representation is needed in order to initiate communication and transfer typed data.

1.2.1 Protocol Types

The following data types are defined and used in protocol packets:

`IMPI_Int4`: 32-bit signed integer

`IMPI_Uint4`: 32-bit unsigned integer

`IMPI_Int8`: 64-bit signed integer

`IMPI_Uint8`: 64-bit unsigned integer

All integral values are in two's complement big-endian format. Big-endian means most significant byte at lowest byte address.

Volume 105, Number 3, May-June 2000
Journal of Research of the National Institute of Standards and Technology

1.2.2 Data Format

The user data transferred is packed at the source and unpacked at the destination using the external data representation "external32" standardized in MPI-2 (section 9.5.2).

1.2.3 Host Identifiers

Host identifiers used in messaging are 16 bytes long. Typically the host IP address is used as the host identifier. A 16-byte container is defined to accommodate the IPv6 protocol.

Advice to implementors. The following text highlights IPv6/IPv4 addressing issues. It is taken from RFC 2373 by R.Hinden & S.Deering:

The IPv6 transition mechanisms include a technique for hosts and routers to dynamically tunnel IPv6 packets over IPv4 routing infrastructure. IPv6 nodes that utilize this technique are assigned special IPv6 unicast addresses that carry an IPv4 address in the low-order 32-bits. This type of address is termed an "IPv4-compatible IPv6 address" and has the format:

80 bits	16 bits	32 bits
0000000000000...................................00000000	000...000	IPv4 Address

A second type of IPv6 address which holds an embedded IPv4 address is also defined. This address is used to represent the address of IPv4-only nodes (those that *do not* support IPv6) as IPv6 addresses. This type of address is termed "IPv4-mapped IPv6 address" and has the format:

80 bits	16 bits	32 bits
0000000000000...................................00000000	111...111	IPv4 Address

(*End of advice to implementors.*)

1.3 Organization of this Document

This document is organized as follows:

- Chapter 2, **Startup/Shutdown**, describes the protocol used to initiate communication.
- Chapter 3, **Data Transfer Protocol**, describes the protocol used to transfer data between two MPI implementations.
- Chapter 4, **Collectives**, specifies the algorithms to be used in collective operations which span multiple MPI implementations
- Chapter 5, IMPI **Conformance Testing**, outlines the preliminary design for a Web-based IMPI conformance testing system.

Chapter 2

Startup/Shutdown

2.1 Introduction

One of the major hurdles to overcome in making different MPI implementations interoperate is launching MPI applications in a multiple-vendor environment. Because we can't encompass all working environments, we must make some basic assumptions about those environments for which interoperability might most reasonably be expected.

ASSUMPTIONS:

1. TCP/IP is available and in use on at least one computer within each implementation universe.

   Rationale. TCP/IP need not necessarily be available on all computers which are to run MPI processes; we merely require that such machines be able to communicate with such a machine running under the local MPI implementation. (*End of rationale.*)

2. The use of rsh must not be assumed. However, all else being equal, those solutions which lend themselves nicely to rsh environments are preferable to those which do not.
3. The use of UNIX must not be assumed. However, all else being equal, those solutions which lend themselves nicely to UNIX environments are preferable to those which do not.

CONCLUSION:

host:port is the best convention to use for establishing initial connections between implementations

2.2 User Steps

2.2.1 Launching A Server

To launch a single job spanning multiple MPI implementations (with a common MPI_COMM_WORLD), a two-step process will be needed in general. The first step is to launch a 'server' process to be used as the rendezvous point for the different implementations. The name of the command used to start IMPI jobs (both the server and client) is implementation dependent; the name `impirun` is used throughout this document to represent this command. Regardless of the actual name, the command must be of the form:

```
impirun -server <count> -port <port_number>
```

Here, <count> is the number of client connections that the server expects to see. When impirun is started with the '-server' option, it creates a TCP/IP socket for listening and then prints both the IP address of the local host (in standard dot notation) and the port number of the socket (to stdout, if on a UNIX machine). If the '-port' option is specified, the server will attempt to start on the given <port_number>. If the '-port' option is not given, the server is free to choose any port number.

Rationale. Printing the complete address instead of only the port number allows for an easy cut-and-paste of the output. And using the IP address instead of the hostname eliminates potential name-lookup problems. (*End of rationale.*)

2.2.2 Launching Clients

```
impirun -client <rank> <host:port> <cmd_line>
```

<rank> specifies where the processes belonging to this client should be placed in MPI_COMM_WORLD relative to the other clients and must be a unique number between 0 and $count - 1$, inclusive.

<host:port> is the host:port string provided by the server.

<cmd_line> is implementation-specific.

2.2.3 Examples

Given a machine named *foo* which will be the server, and two machines named *bar* and *baz* which will be the clients. The user wishes to run 8 copies of a.out on *bar* (with ranks $0 - 7$ in MPI_COMM_WORLD) and 4 copies of b.out on *baz* (with ranks $8 - 11$ in MPI_COMM_WORLD).

On *foo*:
```
impirun -server 2               (typed by user)
128.162.19.8:5678              (output from impirun)
```

On *bar*:
```
impirun -client 0 128.162.19.8:5678 -np 8 a.out
```

On *baz*:
```
impirun -client 1 128.162.19.8:5678 -np 4 b.out
```

Advice to implementors. We do not mandate support for the '-np' syntax, this is simply common practice which we are using for the purpose of example. In general, anything following the host:port argument is completely implementation dependent (and may be quite complex). (*End of advice to implementors.*)

Rationale. The above design allows users with rsh support to write a single shell script to launch a job. For example, on most UNIX systems, the above could be rewritten as follows:

```
#!/bin/csh

setenv hostport 'impirun -server 2 | head -1'

rsh bar impirun -client 0 $hostport -np 8 a.out &
rsh baz impirun -client 1 $hostport -np 4 b.out &

wait
```

On some systems, the above script may only work if impirun is restricted to writing only a single line of text, since subsequent lines could potentially cause a SIGPIPE on impirun after the head process terminates.

A slightly different approach might be to incorporate support for a configuration file directly into the impirun command line:

```
% impirun -server 2 -file appfile
```

where appfile contains:

```
bar -client 0 $hostport -np 8 a.out
baz -client 1 $hostport -np 4 b.out
```

(*End of rationale.*)

2.2.4 Security

Allowing any client on the Internet to establish a connection to the server process may make some users nervous. In light of the fact that security and authentication technology is ever-changing, IMPI is designed to have a modular and upgradable authentication scheme. This scheme is described in Section 2.3.3.

Rationale. Security has become a real concern for all users of the Internet. With the widespread popularity of network scanning tools, an open TCP/IP port on the server node is liable to be discovered by a malicious user, and potentially exploited (especially if the same port is used repeatedly). Many other meta-computing systems offer some form of authentication, ranging from a simple key to more complex protocols to protect against such occurrences. (*End of rationale.*)

2.3 Startup Wire Protocols

2.3.1 Introduction

The IMPI server was designed to be as stupid as possible in order to provide maximum flexibility for future modifications to the clients. Basically it just collects opaque data from each of the clients, concatenates it all together, and broadcasts it back out again.

Each client component of the full job can be broken down into two parts: procs and hosts. Procs are equivalent to MPI processes. Hosts are agents which control a set of procs. Every proc has

exactly one host. A host might have only a single proc or it might have many; this is implementation dependent. For example, when running in a clustered SMP environment, there might reasonably be one host for each machine.

Hosts and procs need not exist on the same physical machine.

2.3.2 The IMPI Server

The server accepts commands of the following form:

```
typedef struct {
      IMPI_Int4 cmd;  // command code
      IMPI_Int4 len;  // length in bytes of command payload
} IMPI_Cmd;
```

The `cmd` field tells the server which command is being sent, and the `len` field tells the server how many bytes of payload are about to follow. In this way, servers can be made forward-compatible by simply discarding any command code that they do not understand.

Note that the traffic in both directions (i.e. client→server and server→client) is always tokenized.

Commands

The following `cmd` values are defined:

```
#define IMPI_CMD_AUTH   0x41555448      // ASCII for 'AUTH'
#define IMPI_CMD_IMPI   0x494D5049      // ASCII for 'IMPI'
#define IMPI_CMD_COLL   0x434F4C4C      // ASCII for 'COLL'
#define IMPI_CMD_DONE   0x444F4E45      // ASCII for 'DONE'
#define IMPI_CMD_FINI   0x46494E49      // ASCII for 'FINI'
```

`AUTH`: pass an authentication key
`IMPI`: setup an IMPI job
`COLL`: collect/broadcast information amongst the IMPI clients
`DONE`: no more COLL labels to submit
`FINI`: all procs have completed (exited) successfully

2.3.3 The `AUTH` command

A client must be authenticated to the server before sending any other commands; the AUTH command *must* be the first command sent to the server. After successful authentication, the client may continue the IMPI startup process. If the authentication is not successful, the server will terminate the client's connection.

> *Advice to implementors.* The protocols that are outlined below, because they are designed to be flexible, may seem somewhat amorphous and non-intuitive when reading. There are comprehensive examples at the end of the description of each authentication protocol. (*End of advice to implementors.*)

The client and server will each have multiple authentication mechanisms available. As such, they must negotiate and agree on a specific method before authentication can proceed. Two authentication mechanisms are currently mandated for all client and server implementations: the IMPI_AUTH_NONE and IMPI_AUTH_KEY protocols.

Rationale. In order to make the authentication mechanism universal between client and server, the list of standardized methods is enumerated below. It is expected that this list can be expanded in the future, both by user requests for specific forms of authentication, and also with advances in authentication technologies. (*End of rationale.*)

Two factors determine whether an authentication method can be used in either the client or the server. First, the mechanism needs to be implemented in the software. Second, the mechanism needs to be enabled by the user. If both of these criteria are met, the authentication method is available for negotiation. If either the client or the server has no authentication mechanisms available for negotiation upon invocation, it will abort with an error message.

Since the client and server may have different authentication mechanisms available for negotiation, they must negotiate to decide on a common method to use. The client begins the negotiation by sending a bit mask of the authentication mechanisms available for negotiation to the server.

```
typedef struct {
     IMPI_Uint4 auth_mask;  // Mask of which authentication
                            // methods the client has available
} IMPI_Client_auth;
```

Advice to implementors. The `auth_mask` is only 32 bits long. While this is probably enough to specify currently available authentication mechanisms, it is possible that it will become desirable to have more than 32 choices in the future. This can be implemented by having the client send multiple `IMPI_Client_auth`'s, and change the value of `len` in the AUTH `IMPI_Cmd` header. (*End of advice to implementors.*)

For each client, the server will compare the client's available methods with its own, and choose the most preferable method that is supported by both. If no common method exists, the server will terminate the connection and display an error message. If a common mechanism exists, the server will inform the client which authentication method it wishes to use, optionally followed by any protocol-specific messages.

```
typedef struct {
      IMPI_Int4 which; // Which authentication will be used
      IMPI_Int4 len;   // Length of follow-on [protocol-specific]
                       // message(s)
} IMPI_Server_auth;
```

The `which` variable is the enumerated value of the authentication mechanism to be used, with the least significant bit of the first `auth_mask` being 0, and the most significant bit being 31. The `len` variable indicates the length of any protocol-specific follow-on message(s) that may be sent by the server immediately after the `IMPI_Server_auth` message. A `len` of zero indicates that the server will not send any protocol-specific messages. All authentication messages after the `IMPI_Server_auth` message are protocol-dependent, and are detailed in the sections below.

The currently supported mechanisms are listed in Table 2.1. Their values are shown with their symbolic (i.e., #define) name, enumerated values (i.e., their corresponding `which` value), and their bit mask form (i.e., their corresponding `auth_mask` value). The symbolic name is synonymous with the enumerated value.

On the command line of the server, the user can specify the order of preference of authentication methods. For example, IMPI_AUTH_NONE (if available for negotiation), should always be last in the order of preference. The following command line syntax will be used to specify the preference list:

Protocol	Enumerated value	Bit mask form
IMPI_AUTH_NONE	0	0x0001
IMPI_AUTH_KEY	1	0x0002

Table 2.1: List of standardized authentication methods, shown in enumerated and bit mask forms.

```
impirun -server N -auth <preference_list>
```

The `<preference_list>` is a comma separated list of `which` value ranges specifying the highest preference on the left. A range can be a single number or a hyphen-separated range of numbers. For example, to specify protocol three as the most preferable, followed by IMPI_AUTH_KEY and IMPI_AUTH_NONE (in that order), the following syntax can be used:

```
impirun -server N -auth 3,1-0
```

If no -auth flag is specified on the command line, the server may choose any authentication mechanism that is available for negotiation on both the client and server.

Advice to implementors. High quality server implementations will choose the "strongest" or "best" form of authentication when multiple authentication mechanisms are available, even if easier, less-secure methods are also available. (*End of advice to implementors.*)

IMPI_AUTH_NONE Protocol

Since some sites can guarantee the security of their networks (behind firewalls, etc.), no authentication is necessary. The IMPI_AUTH_NONE method is designed just for this purpose. The presence of the IMPI_AUTH_NONE environment variable allows the client (and server) to make this method available for negotiation.

If the IMPI_AUTH_NONE protocol is chosen, the `which` value sent to the client will be zero, and the `len` will also be zero. After the server sends the `IMPI_Server_auth` message, the authentication is considered successful; no further authentication messages are sent.

Advice to implementors. Even though the IMPI_AUTH_NONE protocol must be deliberately chosen by the user by setting the IMPI_AUTH_NONE environment variable, it is still a "dangerous" operation. A high quality implementation of the server should warn the user that a client has connected with IMPI_AUTH_NONE authentication by printing a message to the standard output (or standard error), that includes the network address of the connected client. (*End of advice to implementors.*)

Example Authentication Using IMPI_AUTH_NONE

The following command lines show two clients attempting to start. client1 sets the IMPI_AUTH_NONE environment variable and invokes impirun on myprog. client2 does not set the IMPI_AUTH_NONE environment variable, and aborts since the user presumably did not make any other authentication mechanisms available.

```
client1% setenv IMPI_AUTH_NONE
client1% impirun [...client args...] myprog

client2% impirun [...client args...] myprog
Error: No authentication methods available for negotiation.
Aborting.
```

The server also sets the IMPI_AUTH_NONE variable, and invokes impirun. After printing out its IP and socket numbers, it receives a connection from client1, and prints a warning message stating that IMPI_AUTH_NONE was used to authenticate.

```
server% setenv IMPI_AUTH_NONE
server% impirun -server 2
12.34.56.78:9000
Warning: client1.foo.com (12.34.56.78) has authenticated
with IMPI_AUTH_NONE.
```

The messages exchanged by client1 and server were as follows:

```
Client1 sends:  IMPI_Cmd { IMPI_CMD_AUTH, 4 }
Client1 sends:  IMPI_Client_auth { 0x0001 }
Server sends:   IMPI_Server_auth { 0, 0 }
```

At this point, the authentication is considered successful for client1.

IMPI_AUTH_KEY Protocol

The IMPI_AUTH_KEY protocol is a simplistic mechanism that involves the client sending a key to the server. If the client's key matches the server's key, the authentication is successful. If this method of authentication is desired, the value of the key is placed in the IMPI_AUTH_KEY environment variable. The presence of a value in this variable allows both the client and the server to make the IMPI_AUTH_KEY protocol available for negotiation.

If this protocol is chosen, the server sends a `which` value of one, and a `len` of zero back to the client. The client responds with the following message.

```
typedef struct {
      IMPI_Uint8 key; // 64-bit authentication key
} IMPI_Auth_key;
```

If the client's key does not match the key on the server, the server terminates the connection. If the client's key does match, the fact that the server does not terminate the connection indicates a successful authentication.

Example Authentication Using IMPI_AUTH_KEY

The server sets the key value in the environment variable IMPI_AUTH_KEY:

```
server% setenv IMPI_AUTH_KEY 5678
server% impirun -server 2
12.34.56.78:9000
```

The following command lines show two clients attempting to start. client1 sets the IMPI_AUTH_KEY environment variable to the same value as the server, and invokes impirun on myprog. client2 sets the IMPI_AUTH_KEY environment variable to the wrong value, and aborts when the server terminates the connection.

```
client1% setenv IMPI_AUTH_KEY 5678
client1% impirun [...client args...] myprog

client2% setenv IMPI_AUTH_KEY 1234
client2% impirun [...client args...] myprog
Error: Server disconnected (wrong authentication key?)
```

The messages exchanged by the clients and the server are as follows:

```
Client1 sends:  IMPI_Cmd { IMPI_CMD_AUTH, 4 }
Client1 sends:  IMPI_Client_auth { 0x0002 }
Server sends:   IMPI_Server_auth { 1, 0 }
Client1 sends:  IMPI_Auth_key { 5678 }

Client2 sends:  IMPI_Cmd { IMPI_CMD_AUTH, 4 }
Client2 sends:  IMPI_Client_auth { 0x0002 }
Server sends:   IMPI_Server_auth { 1, 0 }
Client2 sends:  IMPI_Auth_key { 1234 }
Server disconnects
```

2.3.4 The `IMPI` command

`IMPI` commands contain the following payload:

```
typedef union {
      IMPI_Int4 rank;       // rank of this client in IMPI job
      IMPI_Int4 size;       // total # of clients in IMPI job
} IMPI_Impi;
```

The `IMPI` command informs the server that this client wishes to join an IMPI job. For the client-to-server packet, the payload consists of the `rank` of the client. After every client in the job has connected to the server and sent its own rank, the server will send back to each client the `size`, that is, the total number of clients in the job.

Example: Consider an IMPI job built from three clients:

client 0 :	3 hosts, each with 2 processes per host
client 1 :	2 hosts, each with 3 processes per host
client 2 :	2 hosts, each with 4 processes per host

The exchange of messages for the `IMPI` command is shown in Figure 2.1. Each client will first send a single `IMPI_Cmd` containing the fields {`IMPI_CMD_IMPI, 4`}. The clients will then each send a single `IMPI_Impi`, with the following fields:

client 0 :	{ `rank = 0` }
client 1 :	{ `rank = 1` }
client 2 :	{ `rank = 2` }

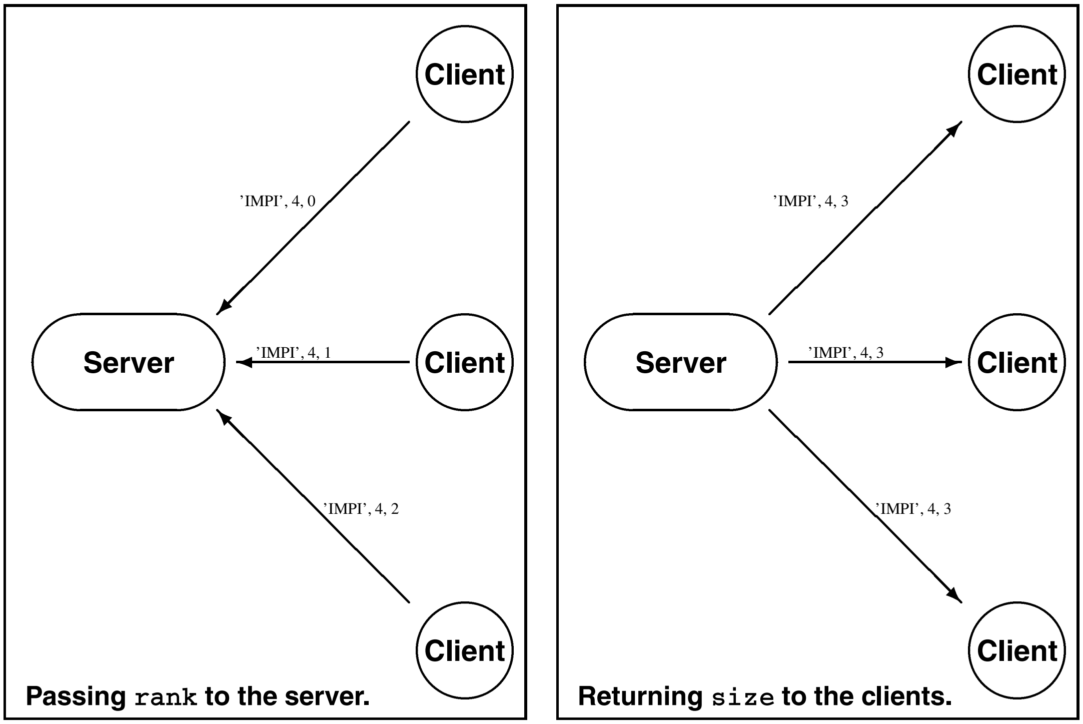

Figure 2.1: `IMPI` command exchange.

After collecting all of the above, the server will send the following `IMPI_Impi` struct back to each client:

```
IMPI_Int4 cmd   = IMPI_CMD_IMPI
IMPI_Int4 len   = 4
IMPI_Int4 size  = 3
```

2.3.5 The `COLL` command

After the server replies to the `IMPI` commands from the clients, it is ready to start collecting other, opaque, startup information from them. This is done via the `COLL` command, which instructs the server to collect one payload from each of the clients and return the concatenation (in ascending client order) of all of them.

All `COLL` payloads sent from the clients to the server begin with the following struct:

```
typedef struct {
      IMPI_Int4 label;
} IMPI_Coll;
```

The `label` field marks the payloads as being of a certain kind; only buffers which share the same label will be concatenated by the server.

All `COLL` payloads sent from the server to the clients begin with the following struct:

```
typedef struct {
      IMPI_Int4 label;
      IMPI_Int4 client_mask;
} IMPI_Chdr;
```

In addition to the `label` field, this `struct` contains the `client_mask` field, which is a bitmask that identifies which clients have submitted values for this label. Bit i in the mask corresponds to the client with rank i (client rank as specified in the `IMPI` command, not an `MPI_COMM_WORLD` rank).

The following `IMPI` labels are currently defined (full explanations are provided below). C++ style comments are used for convenience.

```
#define IMPI_NO_LABEL          0       // reserved for future use

// Per client labels
#define IMPI_C_VERSION         0x1000  // IMPI version(s)
#define IMPI_C_NHOSTS          0x1100  // # of local hosts
#define IMPI_C_NPROCS          0x1200  // # of local procs
#define IMPI_C_DATALEN         0x1300  // maximum data bytes in packet
#define IMPI_C_TAGUB           0x1400  // maximum tag
#define IMPI_C_COLL_XSIZE      0x1500  // coll. crossover size
#define IMPI_C_COLL_MAXLINEAR 0x1600  // coll. crossover # of hosts

// Per host labels
#define IMPI_H_IPV6            0x2000  // IPv6 address
#define IMPI_H_PORT            0x2100  // listening port
#define IMPI_H_NPROCS          0x2200  // # procs per host
#define IMPI_H_ACKMARK         0x2300  // ackmark flow control
#define IMPI_H_HIWATER         0x2400  // hiwater flow control

// Per proc labels
#define IMPI_P_IPV6            0x3000  // IPv6 address
#define IMPI_P_PID             0x3100  // pid
```

The current IMPI version is 0.0. All servers and clients for IMPI version 0.0 must implement all of the above labels, with the exception of `IMPI_NO_LABEL`.

To simplify server implementations, clients are required to pass labels in ascending numeric order. To simplify client implementations, servers are required to broadcast a concatenated buffer as soon as they receive complete sets of buffers from the clients.

Rationale. Ordering these labels allows the server to identify clients that have not implemented a particular label simply by observing the value of the current label sent from that client. Similarly, clients can ignore buffers they receive from the server with labels they do not understand.

The exact order of these labels is not particularly significant except that the `IMPI_C_NHOSTS` label must precede any per-host labels so that clients can correctly interpret the concatenated buffers they receive for the per-host labels. Similarly, the `IMPI_H_NPROCS` label must precede any per-proc label. (*End of rationale.*)

Reserved labels. The following labels are reserved for future use.

IMPI_NO_LABEL This label only exists for future use – it is not used in IMPI version 0.0. It is not sent to the IMPI server.

Client labels. The following labels represent client information.

IMPI_C_VERSION The payload following this label is to comprise of one or more structures of the following type:

```
typedef struct {
      IMPI_Uint4 major;
      IMPI_Uint4 minor;
} IMPI_Version;
```

The client sends an `IMPI_Version` for each version of the IMPI protocols that it supports. All IMPI clients must support version 0.0. The length of the array of `IMPI_Version` structures can be calculated from the payload `len`. Each clients' array of version numbers must be in strictly ascending order.

Each client chooses the highest `major:minor` version number that *all* clients support. Both the major and minor version numbers must match on all clients. This version number determines the nature and content for all future communication between the hosts of each client pair, and may also determine which labels the client will send to the server.

> *Rationale.* Exchanging an IMPI version number between the clients allows for newer protocols to be developed while still maintaining compatibility with older codes. For example, an IMPI version could mandate the minimal set of `IMPI COLL` labels to be recognized.
>
> It is expected that after IMPI version 0.0 begins to be used by real applications, changes in the protocols will be suggested and adopted by the IMPI steering committee. This will change the IMPI version number. The major version number indicates large differences between protocols, while the minor version number indicates smaller changes (such as corrections) in the published protocols.
>
> The IMPI version number should *not* be confused with a particular vendor's software version number. The IMPI version number indicates a published set of protocols, not a particular implementation of those protocols.
>
> Forcing all clients to implement version 0.0 maximizes flexibility and potential for interoperability. (*End of rationale.*)

For example, if the server broadcasts the following data for the `IMPI_C_VERSION` label:

```
IMPI_Int4 cmd         = IMPI_CMD_COLL
IMPI_Int4 len         = 72
IMPI_Int4 label       = IMPI_C_VERSION
IMPI_Int4 client_mask = 0x7
IMPI_Uint4 major      = 0             // from client 0
IMPI_Uint4 minor      = 0
IMPI_Uint4 major      = 0
IMPI_Uint4 minor      = 1
IMPI_Uint4 major      = 0             // from client 1
IMPI_Uint4 minor      = 0
IMPI_Uint4 major      = 0
IMPI_Uint4 minor      = 1
IMPI_Uint4 major      = 0
IMPI_Uint4 minor      = 2
IMPI_Uint4 major      = 0             // from client 2
IMPI_Uint4 minor      = 0
```

```
IMPI_Uint4 major      = 0
IMPI_Uint4 minor      = 1
IMPI_Uint4 major      = 0
IMPI_Uint4 minor      = 2
```

All clients will use IMPI protocol version 0.1 since that is the highest version supported by all clients.

IMPI_C_NHOSTS Each client contributes an `IMPI_Uint4` that indicates the total number of hosts that it has. It must be ≥ 1.

IMPI_C_NPROCS Each client contributes an `IMPI_Uint4` that indicates the total number of procs on all of its hosts. It must be ≥ 1.

IMPI_C_DATALEN Each client contributes an `IMPI_Uint4` that indicates the maximum length, in bytes, of user data in a packet used for host-to-host communication. The smallest value specified by any client determines the value of `IMPI_Pk_maxdatalen` (see section 3.5 Message Packets). It must be ≥ 1.

IMPI_C_TAGUB Each client contributes an `IMPI_Int4` that indicates the maximum tag value that will be used for host-to-host communication. Section 3.9 mandates some restrictions on this value.

IMPI_C_COLL_XSIZE Each client contributes an `IMPI_Int4` that indicates the minimum number of data bytes for which relevant collective calls will use "long" protocols. Relevant collective calls with data sizes less than this value will use "short" protocols.

Clients must provide a way for users to choose this value. If the user does not select a value, the client will contribute -1, indicating that that client wants the default value for this label. If the user does select a value (which must be ≥ 0), that value is sent. All clients must contribute the same value, or an error occurs.

The default value for `IMPI_C_COLL_XSIZE` for IMPI version 0.0 is 1024.

IMPI_C_COLL_MAXLINEAR Each client contributes an `IMPI_Int4` that indicates the minimum number of hosts for which relevant collective calls will use logarithmic protocols. Relevant collective calls with fewer hosts than this value will use linear protocols.

Clients must provide a way for users to choose this value. If the user does not select a value, the client will contribute -1, indicating that that client wants the default value for this label. If the user does select a value (which must be ≥ 0), that value is sent. All clients must contribute the same value, or an error occurs.

The default value for `IMPI_C_COLL_MAXLINEAR` for IMPI version 0.0 is 4.

Host labels. The following labels represent host information.

For each host label, client i contributes an array of `IMPI_C_NHOSTS[i]` values. The array values are ordered; `array[0]` is the value for the lowest numbered host, `array[IMPI_C_NHOSTS[i] - 1]` is the value for the highest numbered host, etc.

The clients read back an array of the collated values; the values for the client 0's hosts begin at index 0, the values for client 1's hosts begin at index `IMPI_C_NHOSTS[0]`, etc.

IMPI_H_IPV6 Each client's array contains the IPv6 addresses for its hosts. Each IPv6 address is a 128 bit quantity (16 bytes).

IMPI_H_PORT Each client's array contains the TCP port numbers for its hosts. Each value is an `IMPI_Uint4`.

IMPI_H_NPROCS Each client's array contains the number of procs on each of its hosts. Each value is an `IMPI_Uint4`.

IMPI_H_ACKMARK Each client's array contains the `IMPI_Pk_ackmark` values for its hosts. Each value is an `IMPI_Uint4`. Section 3.9 mandates some restrictions on this value.

IMPI_H_HIWATER Each client's array contains the `IMPI_Pk_hiwater` values for its hosts. Each value is an `IMPI_Uint4`. Section 3.9 mandates some restrictions on this value.

Proc labels. The following labels represent proc information.

For each proc label, client i contributes an array of `IMPI_C_NPROCS[i]` values. The array values are ordered; `array[0]` is the value for the first proc on the client's first host, `array[IMPI_H_NPROCS[0]]` is the value for the first proc on the client's second host, `array[IMPI_C_NPROCS[i] - 1]` is the value for the last proc on the client's last host, etc.

The clients read back an array of the collated values of length $\sum_{i=1}^{nclients}$`IMPI_C_NPROCS[i - 1]`. The values for the client 0's procs begin at index 0, the values for client 1's procs begin at index `IMPI_C_NPROCS[0]`, etc.

IMPI_P_IPV6 Each client's array contains the IPv6 addresses of the hosts on which each proc resides. Each IPv6 address is a 128 bit quantity (16 bytes).

> *Advice to implementors.* This IPv6 address is only used for unique identification of a proc; it need not be the same as the IPv6 address for the host that the proc resides on. (*End of advice to implementors.*)

IMPI_P_PID Each client's array contains identification numbers for its procs. Each value is an `IMPI_Int8`, and must be unique among other procs that share the same IPv6 address.

Example: per-client labels

Consider the same three-client job from above. After receiving the concatenated `IMPI` buffer from the server, the clients now exchange their startup parameters.

The first parameter is the number of local hosts maintained by each client. In the above example, each client would first send an `IMPI_Cmd` to the server containing {`IMPI_CMD_COLL, 8`}. After the `IMPI_Cmd`, the clients each send the `IMPI_C_NHOSTS` label followed by their local host count. (For a total of 8 bytes, thus the value of 8 in the `len` field of the command.) This exchange is shown in Figure 2.2.

The server, upon receiving all of the `IMPI_C_NHOSTS` data, passes back the concatenated values to the clients:

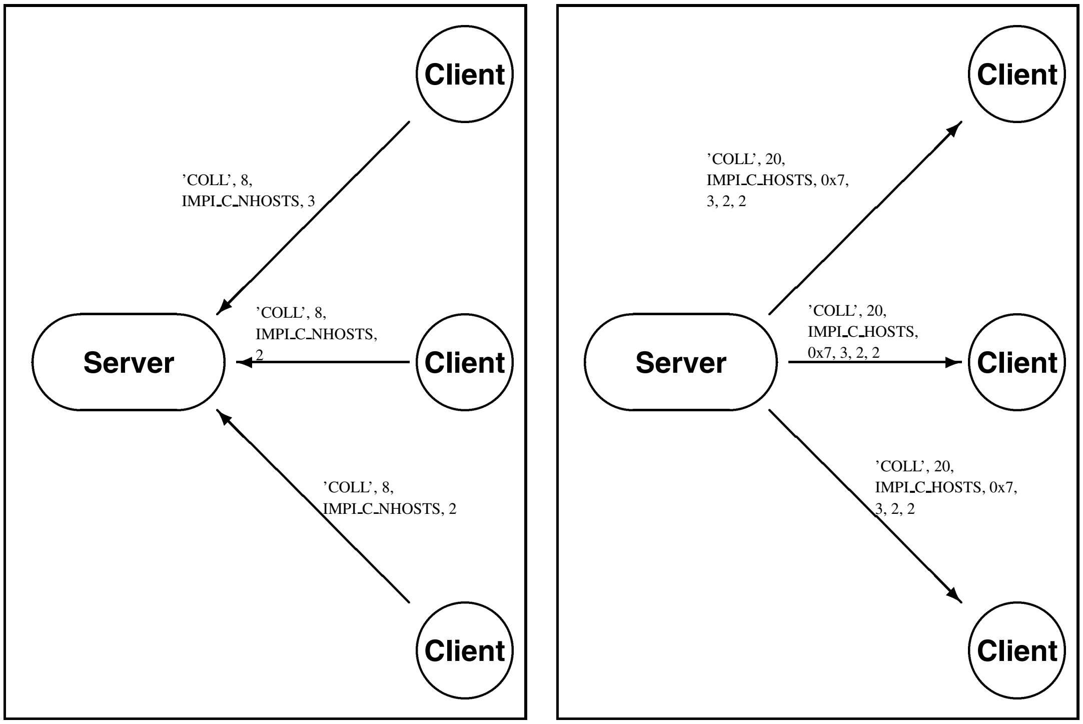

Figure 2.2: `COLL` command `IMPI_C_NHOSTS` exchange.

```
IMPI_Int4 cmd         = IMPI_CMD_COLL
IMPI_Int4 len         = 20
IMPI_Int4 label       = IMPI_C_NHOSTS
IMPI_Int4 client_mask = 0x7
IMPI_Int4 value       = 3             // from client 0
IMPI_Int4 value       = 2             // from client 1
IMPI_Int4 value       = 2             // from client 2
```

All of the per-client values follow this same pattern. For another example, assume that client 0 has a maximum data length of 8000, while clients 1 and 2 have a maximum data length of 4000. They therefore first send an `IMPI_Cmd` to the server containing {`IMPI_CMD_COLL, 8`}. After the `IMPI_Cmd`, the clients each send the `IMPI_C_DATALEN` label followed by their local maximum data length value.

The server, upon receiving all of the `IMPI_C_DATALEN` data, passes back the concatenated values to the clients:

```
IMPI_Int4 cmd         = IMPI_CMD_COLL
IMPI_Int4 len         = 20
IMPI_Int4 label       = IMPI_C_DATALEN
IMPI_Int4 client_mask = 0x7
IMPI_Int4 value       = 8000          // from client 0
IMPI_Int4 value       = 4000          // from client 1
IMPI_Int4 value       = 4000          // from client 2
```

Each client can then independently determine the global minimum data length. Similarly, the `IMPI_C_TAGUB` value will be used by the clients to determine the minimum `tag_ub` among

the clients. The server, being a passive collecting and broadcasting process, knows nothing of the meaning of any of these COLL labels.

Note: Some tags may be optional, and therefore may not be sent by all clients. When this happens, the server will zero the appropriate bit(s) in the client_mask field and just concatenate the values from the participating clients. For example, if client 1 had not passed in a data length, the above would instead have been:

```
IMPI_Int4 cmd         = IMPI_CMD_COLL
IMPI_Int4 len         = 16
IMPI_Int4 label       = IMPI_C_DATALEN
IMPI_Int4 client_mask = 0x5
IMPI_Int4 value       = 8000          // from client 0
IMPI_Int4 value       = 4000          // from client 2
```

This is sent to all clients, regardless of whether they submitted a value for this label. Clients are free to ignore non-mandated COLL labels for the IMPI protocol version(s) that they are using, as well as commands they do not understand.

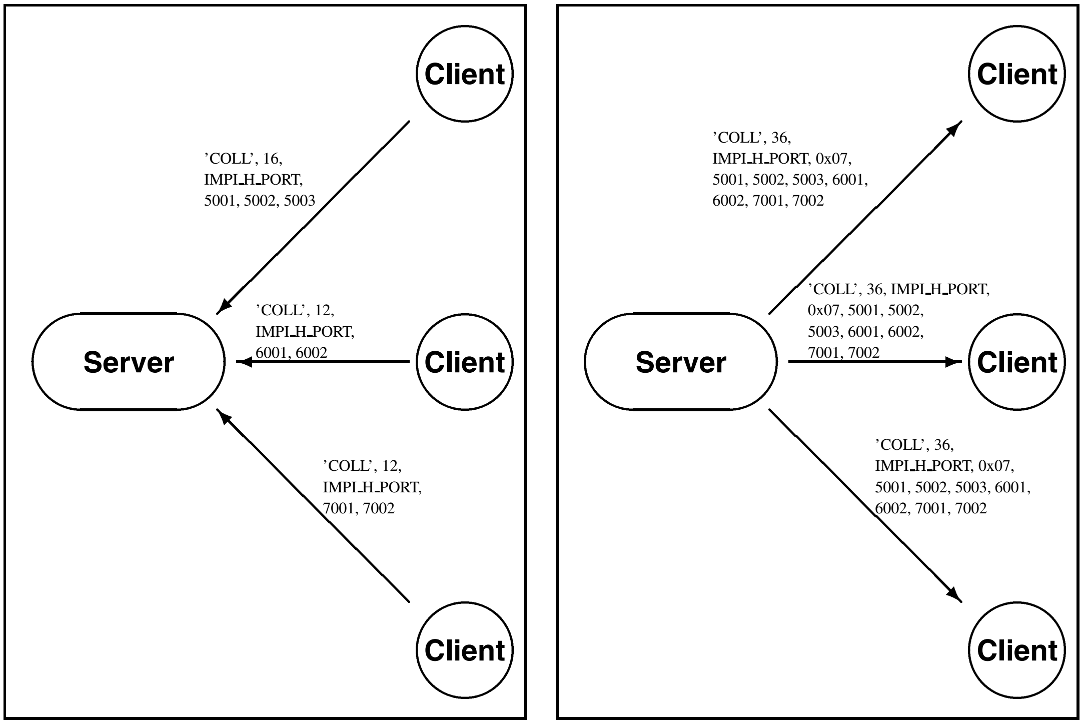

Figure 2.3: COLL command IMPI_H_PORT exchange.

Example: per-host labels

Again using the same three-client example, let's say that it is now time for the clients to submit the port numbers for their hosts. This exchange is shown in Figure 2.3. These would be submitted as follows:

```
client 0:
    IMPI_Int4 cmd         = IMPI_CMD_COLL
    IMPI_Int4 len         = 16
    IMPI_Int4 label       = IMPI_H_PORT
```

```
        IMPI_Int4 port        = 5001 // (or whatever)
        IMPI_Int4 port        = 5002
        IMPI_Int4 port        = 5003

    client 1:
        IMPI_Int4 cmd         = IMPI_CMD_COLL
        IMPI_Int4 len         = 12
        IMPI_Int4 label       = IMPI_H_PORT
        IMPI_Int4 port        = 6001 // (or whatever)
        IMPI_Int4 port        = 6002

    client 2:
        IMPI_Int4 cmd         = IMPI_CMD_COLL
        IMPI_Int4 len         = 12
        IMPI_Int4 label       = IMPI_H_PORT
        IMPI_Int4 port        = 7001 // (or whatever)
        IMPI_Int4 port        = 7002
```

In return, the server would send back:

```
IMPI_Int4 cmd         = IMPI_CMD_COLL
IMPI_Int4 len         = 36
IMPI_Int4 label       = IMPI_H_PORT
IMPI_Int4 client_mask = 0x7
IMPI_Int4 port        = 5001 // from client 0
IMPI_Int4 port        = 5002
IMPI_Int4 port        = 5003
IMPI_Int4 port        = 6001 // from client 1
IMPI_Int4 port        = 6002
IMPI_Int4 port        = 7001 // from client 2
IMPI_Int4 port        = 7002
```

2.3.6 The `DONE` command

This command contains no payload, so it should always have a `len` of zero. It tells the server that a client has no more `COLL` labels to submit. After all clients have issued the `DONE` command, the server sends the `DONE` command, with no payload, back to all clients. This indicates that the startup exchange has completed.

2.3.7 The `FINI` command

Like the `DONE` command, the `FINI` command contains no payload, so it should always have a `len` of zero. The server waits to receive the `FINI` command from all clients. A client must issue the `FINI` command after all of its procs successfully exit. The server does not generate any return traffic to the clients in response to the `FINI` commands. After receiving the `FINI` command from a client the server may close the socket to that client. A server-client socket that dies before the client sends the `FINI` command is an indication of an error which should be reported to the user; server behavior after this error is undefined. The server, after receiving a `FINI` from all clients, exits successful.

Rationale. Leaving the server running while the MPI program is running leaves open the possibility of using it for other purposes such as an error server or a print server. (*End of rationale.*)

2.3.8 Shall We Dance?

The exchange of startup parameters is completed when the DONE command is received from the server. At this point, it may be necessary for additional socket connections to be established. The agents responsible for each socket must therefore participate in a connect/accept "dance", the order of which is defined as follows:

```
/*
 * Higher ranked host connects, lower ranked host accepts.
 */

      for (i = 0; i < nhosts; i++) {
            if (i < myhostrank) {
                  do_connect(i);
            } else if (i > myhostrank) {
                  do_accept();    /* from anybody */
            }
      }
```

After a successful `connect()`, each host must send its rank as a 32-bit value to the accepting process.

2.4 Shutdown Wire Protocols

An IMPI job shuts down in the following order: procs, hosts, clients, and finally, the IMPI server. As per Section 4.24, MPI_FINALIZE invokes a MPI_BARRIER on MPI_COMM_WORLD. After this barrier, each proc performs implementation-specific cleanup and shutdown.

After all the procs of a host have completed (meaning that there will be no further MPI communications from each proc), the host will send an IMPI_PK_FINI packet to each other host (see Section 3.8), indicating that it is shutting down. The host must wait for the corresponding IMPI_PK_FINI from each other host before closing a communications channel. The host must consume arriving packets and issue appropriate acknowledgments until the IMPI_PK_FINI arrives. The host then performs implementation-specific cleanup and shutdown.

After all the hosts of a client have completed (meaning that all IMPI_PK_FINI messages have been sent, and all of the client's host communication channels have been closed), the client sends an IMPI_CMD_FINI message to the IMPI server. The client then performs implementation-specific cleanup and shutdown.

After the IMPI server receives an IMPI_CMD_FINI message from each client, it performs its own cleanup and shutdown.

Rationale. The sequence of events during IMPI shutdown is mandated to avoid race conditions and deadlock. (*End of rationale.*)

2.5 Client and Host Attributes

This section is heavily influenced by Section 5.3 of the MPI-2 Journal of Development, "Cluster Attributes."

Inter-client and inter-host communications may be significantly slower than communications between processes on the same host. It is therefore desirable for programs to be able to determine which ranks are local and which are remote.

The following attributes are predefined on all communicators:

IMPI_CLIENT_SIZE returns as the keyvalue the number of IMPI clients included in the communicator.

IMPI_CLIENT_COLOR returns as the keyvalue a number between 0 and IMPI_CLIENT_SIZE-1. The value returned indicates with which client the querying rank is associated. The relative ordering of colors corresponds to the ordering of host ranks in MPI_COMM_WORLD.

IMPI_HOST_SIZE returns as the keyvalue the number of IMPI hosts included in the communicator.

IMPI_HOST_COLOR returns as the keyvalue a number between 0 and IMPI_HOST_SIZE-1. The value returned indicates with which client the querying rank is associated. The relative ordering of colors corresponds to the ordering of host ranks in MPI_COMM_WORLD.

Advice to users. This interface returns no information about the significance of the difference between the communication *inside* and *between* client/hosts members. However, this can be achieved by small application-specific benchmarks as part of the application. The returned color can be used as input to IMPI_COMM_SPLIT. (*End of advice to users.*)

Chapter 3

Data Transfer Protocol

3.1 Introduction

This chapter specifies the protocol used to transfer data between two MPI implementations. The protocol assumes a reliable, ordered, bidirectional stream communication channel between the two implementations. The channel is assumed to have a finite but unspecified amount of buffering. The protocol does not rely on the channel buffering for its operation. Processes on one side of the channel belong to the same MPI implementation. Two implementations communicate via a dedicated channel. Message routing (the selection of a channel to use for a particular message transfer) is not addressed here. It is assumed that an agent at the source determines the appropriate channel to use and directs the message to it. In essence, the data transfer protocol enables multiple processes to have timeshared access to a single communication channel, and provides mechanisms to throttle fast senders and cancel transferred messages.

The protocol is defined independently of the underlying channel technology. Initially, TCP/IP is expected to be used by most implementations. Some implementations may opt for a restricted interoperability space and choose a different channel technology, while others may support multiple technologies. The protocol does not specify the interaction between processes and their agent nor the medium used (e.g. sockets, shared-memory). To provide generality of implementation, no restrictions are placed on the process/agent setup (e.g. shared access to socket, file descriptor passing). To support the MPI-2 client/server functionality, no parent/child relationship is assumed between processes and their agent.

3.2 Process Identifier

Messages exchanged between implementations are multiplexed in the channel. A system-wide unique process identifier is required to label the message source and destination. To support the MPI-2 client/server functionality, a decentralized mapping of processes to identifiers is chosen. The `IMPI_Proc` process identifier is defined as the combination of a system-wide unique host identifier and a process identifier unique within the host:

```
typedef struct {
      IMPI_Uint1           p_hostid[16]; /* host identifier */
      IMPI_Int8            p_pid;        /* local process identifier */
} IMPI_Proc;
```

Typically, the host IP address is used as host identifier. A 16-byte container is defined to

accommodate the IPv6 protocol.

Solutions with a restricted interoperability scope may select other host identification methods. IMPI does not mandate p_pid to be unique across all implementations within a given host. Thus IMPI does not guarantee interoperability between two implementations that share a host within a single MPI application.

> *Advice to implementors.* Implementors are encouraged to use an OS-wide unique p_pid identifier within a host, such as a UNIX pid. This would support IMPI host sharing in practice, and can be helpful for situations such as testing IMPI functionality. (*End of advice to implementors.*)

3.3 Context Identifier

A context identifier of type IMPI_Uint8 is associated with every MPI communicator (intra- and inter-communicators). It has the following properties:

- It uniquely identifies a communicator within a process.
- All processes within a communicator group use the same context identifier for that communicator.

The context identifier of MPI_COMM_WORLD is 0.

> *Advice to implementors.* Mandating a collectively unique context ID may be a burden on some implementations that use memory addresses to segregate message contexts. Such implementations may choose to let the agent handle the mapping between context IDs and memory addresses and not impact the performance of the intra-implementation communication protocols. (*End of advice to implementors.*)

3.4 Message Tag

The message tag is of type IMPI_Int4. MPI requires MPI_TAG_UB to be at least 32767. At startup time, the actual tag upper bound, IMPI_Tag_ub, is negotiated between the implementations.

3.5 Message Packets

MPI requires that messages of active requests be uniquely identified to allow for their cancellation. Requests that have been completed or are otherwise inactive cannot be canceled. As a result, an IMPI message is identified by its source and destination processes, and by a source request identifier unique for every active request at the source process. The request identifier is of type IMPI_Uint8. The total message length is represented by a value of type IMPI_Uint8. The sender's local rank in the communicator is given in the pk_lsrank field of the header. This allows the receiving process to set the MPI_SOURCE status entry without having to map pk_src to its local rank.

> *Advice to implementors.* On systems with 64-bit memory addressing or less, the address of the request object at the source process may be used as the unique identifier of an active request. On systems with wider memory addressing, the source process would need to maintain a mapping of active requests to identifiers.

The source and destination processes are identified by their IMPI_Proc structures instead of their local ranks in the communicator used. This gives implementors more freedom in the design of the internal agent protocol with respect to message buffering and matching messages to receive requests. For example, messages may be buffered and matched:

- in the agent, the agent acting as an MPI-aware "database".
- in the receiving processes, the agent acting purely as a funnel.
- a mixture of both where the agent handles the buffering and matching on a destination process basis, and the receiving processes handle the buffering and matching of MPI tags and context IDs.

(*End of advice to implementors.*)

A message is divided into packets, each containing up to IMPI_Pk_maxdatalen bytes of user data. The IMPI_Pk_maxdatalen value is negotiated at startup time. All message packets are sent in the same channel, in sequential order. A fixed-length packet header, IMPI_Packet, holds the message information and identifies the type of transfer: data packet (synchronous message or not), synchronization or protocol acknowledgment (ACK), cancel request, cancel reply (successful or not), or finalization. The maximum size of a packet is IMPI_Pk_maxdatalen plus the size of the IMPI_Packet header.

In addition to identifying messages for cancellation, the source request identifier is used by the sender to access the request handle in the rendezvous message protocol. Similarly, an optional destination request identifier, of type IMPI_Uint8, may be used to accelerate the receiving process's access to the request handle. Its usage and the required support by the peer agent is discussed in a later section.

Three optional quality-of-service fields are made available. They may be used by collaborating implementations to provide additional services, such as profiling or debugging. If used, pk_count holds the message "count" argument (number of datatype elements), pk_dtype is an opaque handle that uniquely identifies the sender's datatype within the process (e.g. the handle of the datatype object), and pk_seqnum holds a sequence number that helps identify a message independently of its source request ID, which may be reused (e.g. a sequence number unique per sending process, per sending agent, per sender/receiver process pair, or per agent pair).

```
typedef struct {
        IMPI_Uint4           pk_type;      /* packet type */
#define IMPI_PK_DATA         0             /* message data */
#define IMPI_PK_DATASYNC     1             /* message data (sync) */
#define IMPI_PK_PROTOACK     2             /* protocol ACK */
#define IMPI_PK_SYNCACK      3             /* synchronization ACK */
#define IMPI_PK_CANCEL       4             /* cancel request */
#define IMPI_PK_CANCELYES    5             /* 'yes' cancel reply */
#define IMPI_PK_CANCELNO     6             /* 'no' cancel reply */
#define IMPI_PK_FINI         7             /* agent end-of-connection */

      IMPI_Uint4           pk_len;      /* packet data length */
      IMPI_Proc            pk_src;      /* source process */
      IMPI_Proc            pk_dest;     /* destination process */
      IMPI_Uint8           pk_srqid;    /* source request ID */
      IMPI_Uint8           pk_drqid;    /* destination request ID */
      IMPI_Uint8           pk_msglen;   /* total message length */
```

```
    IMPI_Int4            pk_lsrank;   /* comm-local source rank */
    IMPI_Int4            pk_tag;      /* message tag */
    IMPI_Uint8           pk_cid;      /* context ID */
    IMPI_Uint8           pk_seqnum;   /* message sequence # */
    IMPI_Int8            pk_count;    /* QoS: message count */
    IMPI_Uint8           pk_dtype;    /* QoS: message datatype */
    IMPI_Uint8           pk_reserved; /* for future use */
} IMPI_Packet;
```

Advice to implementors. The choice of 4- or 8-byte integers in IMPI_Packet is a trade-off between providing enough storage space where needed with some room for future extensions, keeping the structure size a power-of-two value (128 bytes in this case), and ordering the elements to avoid compiler padding. (*End of advice to implementors.*)

The data packet is made of a header followed by up to IMPI_Pk_maxdatalen bytes of packed user data, and uses all the header fields. The four other packet types are header-only, and use a subset of the header fields. The list of fields used by each packet is given in table 3.1. Network byte-order is used in the header.

3.6 Packet Protocol

At the packet level, a simple throttling protocol is setup to limit the amount of buffering required and to prevent fast senders from affecting the message flow of other processes sharing the channel. This creates process-pair virtual channels. The number of virtual channels mapped onto a single channel is not fixed and can change according to the application's behavior. The communication agents are expected to handle the resulting change in buffering requirement. At startup time, two packet protocol values of type IMPI_Uint4 are negotiated:

IMPI_Pk_ackmark: The number of packets received by the destination process before a protocol ACK is sent back to the source.

IMPI_Pk_hiwater: The maximum number of unreceived packets the source can send before requiring a protocol ACK to be send back.

For each process-pair, the source maintains a packets-sent counter and the destination maintains a packets-received counter. The destination process sends a protocol ACK to the source process for every IMPI_Pk_ackmark packets it receives from that source. This decrements the

Packet Type	Fields Used
data	all fields (QoS fields optional)
data sync.	all fields (QoS fields optional)
sync. ACK	pk_type, pk_src, pk_dest, pk_srqid, pk_drqid
protocol ACK	pk_type, pk_src, pk_dest
cancel request	pk_type, pk_src, pk_dest, pk_srqid
cancel reply	pk_type, pk_src, pk_dest, pk_srqid
finalization	pk_type

Table 3.1: Packet field usage.

source's counter by `IMPI_Pk_ackmark`. When the source's counter reaches the `IMPI_Pk_hiwater` value, it refrains from sending more packets to that destination until an ACK is received from it. The transfer of protocol ACK packets does not modify the value of the counters. The implementation is expected to provide sufficient buffering to receive the protocol ACK packets and to expedite their processing.

> *Advice to implementors.* Depending on the implementation's internal process/agent protocol, packet counters can either be maintained by the processes or by the agent. (*End of advice to implementors.*)

3.7 Message Protocols

The data, synchronization, cancel request, and cancel reply packet types are used to construct the protocols for handling MPI point-to-point transfers. A message with length of up to `IMPI_Pk_maxdatalen` bytes is categorized as a short message. It fits in a single data packet. Longer messages are split into several data packets. The IMPI_PK_DATASYNC packet type notifies the receiving process that the sender is expecting a synchronization ACK for the message. Otherwise, the IMPI_PK_DATA packet type is used.

3.7.1 Short-Message Protocol

Short messages are sent eagerly, relying on the packet protocol ACKs for flow control. The `pk_len` and `pk_msglen` fields have the same value. If the IMPI_PK_DATASYNC packet type is used, the destination process sends a synchronization ACK packet back to the source after it matches the message to a receive request.

The `pk_srqid` field in the ACK packet must be set to the value of the `pk_srqid` field in the message packet. The sender must store the send request identifier in the outgoing packet. It receives it back in the ACK packet. This mechanism is used by the sending process to locate the request that matches the ACK packet.

Short messages generated by MPI_SSEND and MPI_ISSEND are mapped onto the short-message protocol with the IMPI_PK_DATASYNC packet type. All other short messages are mapped onto this protocol with the IMPI_PK_DATA packet type.

3.7.2 Long-Message Protocol

For long messages, the first data packet is sent eagerly, with the IMPI_PK_DATASYNC packet type. When the destination process matches the packet to a receive request, it sends a synchronization ACK packet back to the source process. The source can then send all remaining data packets with the IMPI_PK_DATA packet type.

The `pk_srqid` field in the ACK packet must be set to the value of the `pk_srqid` field in the message packet. The sender must store the send request identifier in the outgoing packet. It receives it back in the ACK packet. This mechanism is used by the sending process to locate the request that matches the ACK packet.

Likewise, the `pk_drqid` field in the data packets sent after the ACK packet is received (that is all data packets except the first one) must be set to the value of the `pk_drqid` field in the ACK packet. The receiving process may use this field to store a handle to the matching MPI receive

request in the ACK packet, and receive it back in all the following data packets. This avoids having the receiving process search for the matching request for each remaining data packet.

Long messages generated by all MPI send calls are mapped onto the long-message protocol, independent of their blocking nature and synchronization requirements.

3.7.3 Message-Probing Protocol

Supporting the MPI_PROBE and MPI_IPROBE functions does not require special packet transfers. The protocol is purely local between the process and the agent. IMPI defines the conditions under which a message is considered to be available for the purpose of probing.

Packet transfers are considered atomic operations, independent of the medium's transfer mechanism. A message is considered available to the destination process after its first packet (its only packet for short messages) has been completely read by the agent, including the packet's user data segment. The total length of the message is available in the packet's `pk_msglen` field. Thus the status information needed by the probe calls is available.

3.7.4 Message-Cancellation Protocol

For a send request, there is a time window during which a call to MPI_CANCEL can cause a cancel request packet to be sent to the message destination. This can happen in the following cases:

- After a short non-synchronous message is sent.
- After a short synchronous message is sent and before the synchronization ACK is received.
- After the first packet of a long message is sent and before the synchronization ACK is received.

In all other cases, the MPI_CANCEL call must be resolved locally. Once a cancel request is sent, a cancel reply packet must be returned, independently of whether a synchronization ACK for that message was already sent back. This allows MPI_CANCEL to act as a simple RPC call, waiting for the reply, and simplifies the operations of the agents. If the message's first data packet has not been received by the destination process (i.e. matched a receive request), the agent sends a IMPI_PK_CANCELYES reply packet and atomically destroys the buffered packet. Otherwise, a IMPI_PK_CANCELNO packet is sent back. Note that due to the message ordering guarantee, a cancel request cannot be received without the agent having fully read the message's first packet. Thus the message can be in either of two states: buffered, or received by the destination process. The transition from a buffered state to a received state happens when the message's first packet matches a receive request, irrespective of the state of the remaining packets in the case of a long message.

For a given pair of processes, the sender's request identifier is used by the receiver to select the message to be canceled. The request identifier is unique among the sender's active requests. It is possible that multiple messages buffered at the receiver share the same sender request identifier. In such a case, only the last message received can be canceled, the other messages are no longer attached to the active request. This requires that the storage of unexpected messages be searchable in reverse chronological order.

3.8 Finalization Protocol

When an agent determines that its processes no longer require a channel for communication, it sends a finalization packet (IMPI_PK_FINI packet type) to notify the agent on the opposite side of the channel of its request to terminate the connection. An agent may not close the connection until it has sent an IMPI_PK_FINI packet to the other agent and received one from it. Until a finalization packet is received, an agent must continue to consume arriving packets and issue the appropriate acknowledgments; this effectively destroys unmatched messages. Acknowledgment are the only packets an agent may send on a channel after it issues a finalization packet. Because the finalization packet is exchanged between agents, it does not require buffering and thus does not affect the protocol ACK counters. The finalization protocol allows agents to distinguish between applications that terminate successfully and those that terminate abnormally (see section 2.4). It does not mandate error handling for the latter.

> *Advice to implementors.* The protocol does not specify how an agent determines when its processes no longer need the channel. This is a local implementation-specific synchronization between MPI_FINALIZE and the agent.
>
> It is recommended that the agent not set the TCP/IP socket's SO_LINGER option to a linger time of zero. If it is set to zero, the connection may be destroyed before the IMPI_PK_FINI packet reaches its destination. This causes the receiving agent to erroneously conclude that the application terminated abnormally.
>
> The protocol does not specify the error handling an agent performs in cases of unmatched messages. It only requires that unmatched messages be destroyed. (*End of advice to implementors.*)

3.9 Mandated Properties

To support wide interoperability, IMPI requires the data transfer channel to be an Internet-domain socket. In addition, the following must be true:

- $1 \leq$ `IMPI_Pk_ackmark` $\leq$ `IMPI_Pk_hiwater`
- $1 \leq$ `IMPI_Pk_maxdatalen`
- $32767 \leq$ `IMPI_Tag_ub` ≤ 2147483647

Chapter 4

Collectives

4.1 Introduction

This chapter specifies the algorithms to be used in collective operations on communicators which span multiple MPI clients.

A *native communicator* is defined to be a communicator in which all processes are running under the same MPI client.

IMPI places no restrictions on and does not specify the implementation of collective operations on native communicators.

Processes running under the same MPI client are defined to be *local* processes. Similarly communication between between processes running under the same MPI client is called *local* communication.

Communication between processes running under different MPI clients is referred to as *non-local* or *global*.

Many of the collective operations consist of one or more local and global phases of communication. An IMPI implementation is free to implement local phases in whatever manner it chooses but must implement the global phases as specified in order to properly interoperate with other implementations. No global communications may be done other than those explicitly specified.

In the specifications of the collectives great liberties have been taken with the cleaning up of temporary objects, e.g. intermediate groups. It is expected that implementors will add the necessary resource freeing. Additionally little specific error handling is specified. It is expected that implementors will check the return codes of MPI functions used and return appropriately on error.

Some of the collective operations require that data packed by one implementation be unpacked by another implementation. This requires that all implementations use the same format for such packed data. This leads to the following restriction on MPI_Pack() in the case of non-native communicators. The format of data packed by a call to MPI_Pack() with a non-native communicator is the wire (external32) format with no header or trailer bytes. In addition a call to MPI_Pack_size() with a non-native communicator will return as the size the minimum number of bytes required to represent the data in the wire (external32) format.

No restriction is placed on the behavior of MPI_Pack() and MPI_Pack_size() when called with native communicators.

4.2 Utility functions

For a given communicator there is one *master* process per MPI client the communicator spans. The master process for a client is the process of lowest rank running under the client. Note that process rank 0 within a communicator is always a master process. The master processes in a communicator are numbered from 0 to (number of masters) -1 in order of rank in the communicator.

For example consider the case of a communicator of size 8 which spans 3 clients (say A, B and C) with ranks 0,1,4 under client A, ranks 2,3,5 under client B and ranks 6,7 under client C. Then the master processes are ranks 0,2 and 6 and they are numbered 0,1 and 2 respectively.

The descriptions of the IMPI collectives make use of the following utility functions. Each implementation is free to implement them in whatever manner they see fit.

int is_master(int r, MPI_Comm comm) Returns TRUE iff process rank r in comm is a master process.

int are_local(int r1, int r2, MPI_Comm comm) Returns TRUE iff processes ranked r1 and r2 in comm are local to one-another.

int master_num(int r, MPI_Comm comm) If process rank r in comm is a master process returns its master number else returns -1.

int master_rank(int n, MPI_Comm comm) Returns the rank in comm of master number n.

int local_master_num(int r, MPI_Comm comm) Returns the master number of the master process local to process rank r in comm.

int local_master_rank(int n, MPI_Comm comm) Returns the rank in comm of the master process local to process rank r in comm.

int num_masters(MPI_Comm comm) Returns the number of master processes in comm.

int num_local_to_master(int n, MPI_Comm comm) Returns the number of processes in comm local to master process number n.

int num_local_to_rank(int r, MPI_Comm comm) Returns the number of processes in comm local to process rank r in comm.

int *locals_to_master(int n, MPI_Comm comm) Returns an array containing the ranks in comm of the processes local to master number n.

int cubedim(int n) If $n > 0$ returns the dimension of the smallest hypercube containing at least n vertices (i.e. smallest i such that $n <= 2^i$) else returns -1.

int highbit(int r, int dim) Returns the position of the highest bit set in the lowest dim bits of r else -1 if no bit is set in the lowest dim bits of r. E.g. highbit(5,3) = 2, highbit(5,2) = 0, highbit(8,2) = -1.

4.3 Context Identifiers

Context IDs are of type `IMPI_Uint8` and are collectively unique. Collectively unique means the context IDs for a communicator are the same for each process in the communicator and no other communicator of which the process is a member has the same context IDs.

> *Advice to implementors.* Mandating collectively unique context IDs may be a burden on some implementations that use memory addresses to segregate message contexts. Such implementations may choose to let the agent handle the mapping between context IDs and memory addresses and not impact the performance of the intra-implementation communication protocols. (*End of advice to implementors.*)

Each communicator has two context IDs. One is used for point-to-point communication and the other for collective communication. The collective context ID is always one greater than the point-to-point context ID.

The point-to-point context identifier of MPI_COMM_WORLD is 0 and the collective context identifier is 1.

Many of the collective operations are defined in terms of point-to-point communications on a communicator. All point-to-point communications which occur inside collectives must use the communicator's collective context ID.

> *Advice to implementors.* This can be done, for example, by passing a shadow "collective version" of the communicator to the point-to-point communication. (*End of advice to implementors.*)

4.3.1 Context ID Creation

When a new communicator is created it must be assigned collectively unique context IDs. Generating the new context ID is a collective operation over the communicator(s) from which the new one is being derived.

The basic mechanism for creating a new context ID is to first find the maximum context ID currently in use by any process involved in the new context creation. The new point-to-point context ID is then this maximum plus one and the new collective context ID is this maximum plus two.

In the descriptions of the collective algorithms which create new contexts it is assumed that each process keeps track of the maximum context ID it has in use in the variable

```
IMPI_Uint8 IMPI_max_cid;
```

which is initialized to 1 in `MPI_Init()`.

> *Advice to implementors.* Implementations are free to allocate other context IDs (e.g. for shadow communicators) but they must ensure that the value of IMPI_max_cid is correctly maintained.
>
> In systems with limited context ID space the agent for each process can maintain a mapping between the limited space and the 64-bit IMPI space. (*End of advice to implementors.*)

4.4 Comm_create

```
int MPI_Comm_create(MPI_Comm comm, MPI_Group group, MPI_Comm *newcomm)
{
  IMPI_Uint8 newcid, maxcid;

  /* create a new context ID */
  if (IMPI_max_cid % 2 == 0) IMPI_max_cid += 1;
  MPI_Allreduce(&IMPI_max_cid, &maxcid, 1, IMPI_UINT8, MPI_MAX, comm);
  if (maxcid >= IMPI_MAX_CID-1)
    error out of contexts;

  newcid = maxcid + 1;
  IMPI_max_cid = maxcid + 2;

  build a new communicator newcomm from group with point-to-point
  context newcid and collective context (newcid+1);
}
```

4.5 Comm_free

```
int MPI_Comm_free(MPI_Comm *comm)
{
  if (*comm is an intra-communicator) {
    MPI_Barrier(*comm);
  } else {
    /* Inter-communicator, cannot use collection operations.
     * Perform a barrier with point-to-point calls only.
     */
    int i, myrank, rgsize;
    MPI_Status status;

    MPI_Comm_remote_size(*comm, &rgsize);
    MPI_Comm_rank(*comm, &myrank);

    /* Fan-in from local ranks to remote rank 0. */
    if (myrank == 0) {
      for (i=1; i<rgsize; i++) {
        MPI_Recv(MPI_BOTTOM, 0, MPI_BYTE, i, IMPI_COMM_FREE_TAG,
                 *comm, &status);
      }
    } else {
      MPI_Send(MPI_BOTTOM, 0, MPI_BYTE, 0, IMPI_COMM_FREE_TAG, *comm);
    }

    /* Swap between local and remote rank 0's. */
    if (myrank == 0)
      MPI_Sendrecv(MPI_BOTTOM, 0, MPI_BYTE, 0, IMPI_COMM_FREE_TAG,
                   MPI_BOTTOM, 0, MPI_BYTE, 0, IMPI_COMM_FREE_TAG,
                   *comm, &status);

    /* Fan-out from local rank 0 to remote group. */
```

```
    if (myrank == 0) {
      for (i=1; i<rgsize; i++)
        MPI_Send(MPI_BOTTOM, 0, MPI_BYTE, i, IMPI_COMM_FREE_TAG, *comm);
    } else {
      MPI_Recv(MPI_BOTTOM, 0, MPI_BYTE, 0, IMPI_COMM_FREE_TAG,
               *comm, &status);
    }
  }

  mark communicator with handle *comm for deallocation;
  *comm = MPI_COMM_NULL;
  return MPI_SUCCESS;
}
```

MPI_Comm_free merely marks a communicator for deallocation and does not necessarily immediately deallocate it. When the communicator is actually deallocated its context IDs are freed. Implementations may keep track of the context IDs which are in use and lower IMPI_max_cid appropriately when freeing a context ID.

4.6 Comm_dup

```
int MPI_Comm_dup(MPI_Comm comm, MPI_Comm *newcomm)
{
  MPI_Status  status;
  IMPI_Uint8  newcid, maxcid;
  MPI_Group   group;
  MPI_Comm    localcomm, tmpcomm;
  int         myrank, rgsize;

  MPI_Comm_rank(comm, &myrank);

  /* create a new context ID */
  if (IMPI_max_cid % 2 == 0) IMPI_max_cid += 1;

  if (comm is an intra-communicator) {
    MPI_Allreduce(&IMPI_max_cid, &maxcid, 1, IMPI_UINT8, MPI_MAX, comm);
  } else {
    /* Rank 0 processes are the leaders of their local group.
     * Each leader finds the max context ID of all remote group
     * processes (excluding their leader).  The leaders then swap the
     * information and broadcast to the remote group.
     */
    MPI_Comm_remote_size(comm, &rgsize);

    if (myrank == 0) {
      maxcid = IMPI_max_cid;

      /* find max context ID of remote non-leader processes */
      for (i = 1; i < rgsize; i++) {
        MPI_Recv(&newcid, 1, IMPI_UINT8, i, IMPI_DUP_TAG, comm, &status);
        if (newcid > maxcid) maxcid = newcid;
      }
```

```
      /* swap context ID with remote leader */
      MPI_Sendrecv(&maxcid, 1, IMPI_UINT8, 0, IMPI_DUP_TAG,
                   &newcid, 1, IMPI_UINT8, 0, IMPI_DUP_TAG,
                   comm, &status);

      if (newcid > maxcid) maxcid = newcid;

      /* broadcast context ID to remote non-leader processes */
      for (i = 1; i < rgsize; i++)
        MPI_Send(&maxcid, 1, IMPI_UINT8, i, IMPI_DUP_TAG, comm);
    }
    else {
      /* non-leader */
      MPI_Send(&IMPI_max_cid, 1, IMPI_UINT8, 0, IMPI_DUP_TAG, comm);
      MPI_Recv(&maxcid, 1, IMPI_UINT8, 0, IMPI_DUP_TAG, comm, &status);
    }
  }

  if (maxcid >= IMPI_MAX_CID-1)
    error out of contexts;

  newcid = maxcid + 1;
  IMPI_max_cid = maxcid + 2;

  build a new communicator newcomm with the same groups as comm and
    with point-to-point context newcid and collective context (newcid+1)

  return MPI_SUCCESS;
}
```

4.7 Comm_split

```
int MPI_Comm_split(MPI_Comm comm, int color, int key, MPI_Comm *newcomm)
{
  int *p, *p2, *procs;
  int nprocs, myrank, *myprocs, mynprocs;
  MPI_Group oldgroup, newgroup;
  IMPI_Uint8 newcid, maxcid;

  /* create a new context ID */
  if (IMPI_max_cid % 2 == 0) IMPI_max_cid += 1;

  MPI_Allreduce(&IMPI_max_cid, &maxcid, 1, IMPI_UINT8, MPI_MAX, comm);
  if (maxcid >= IMPI_MAX_CID-1)
    error out of contexts;

  newcid = maxcid + 1;
  IMPI_max_cid = maxcid + 2;

  /* create an array of process information for doing the split */
  MPI_Comm_size(comm, &nprocs);
  MPI_Comm_rank(comm, &myrank);
```

```
  procs = (int *) malloc(3 * nprocs * sizeof(int));

  /* gather all process information at all processes */
  p = &procs[3 * myrank];
  p[0] = color;
  p[1] = key;
  p[2] = myrank;

  MPI_Allgather(p, 3, MPI_INT, procs, 3, MPI_INT, comm);

  /* processes with undefined color can stop here */
  if (color == MPI_UNDEFINED) {
    *newcomm = MPI_COMM_NULL;
    return MPI_SUCCESS;
  }

  sort the array of process information in ascending order by
    color, then by key if colors are the same, then by rank if color
    and key are the same;

  /* locate and count the # of processes having my color */
  myprocs = 0;
  for (i = 0, p = procs; (i < nprocs) && (*p != color); ++i, p += 3);

  myprocs = p;
  mynprocs = 1;

  for (++i, p += 3; (i < nprocs) && (*p == color); ++i, p += 3) {
      ++mynprocs;
  }

  /* compact the old ranks of my old group in the array */
  p = myprocs;
  p2 = myprocs + 2;
  for (i = 0; i < mynprocs; ++i, ++p, p2 += 3) *p = *p2;

  /* create the new group */
  MPI_Comm_group(comm, &oldgroup);
  MPI_Group_incl(oldgroup, mynprocs, myprocs, &newgroup);
  MPI_Group_free(&oldgroup);

  create a new communicator newcomm with point-to-point context newcid,
    collective context (newcid+1) and group newgroup;

  return MPI_SUCCESS;
}
```

4.8 Intercomm_create

```
int MPI_Intercomm_create(MPI_Comm lcomm, int lleader, MPI_Comm pcomm,
                         int pleader, int tag, MPI_Comm *newcomm)
{
```

```
MPI_Status status;
MPI_Group oldgroup, remotegroup;
IMPI_Uint8 newcid, maxcid;
IMPI_Uint8 inmsg[2], outmsg[2];
int lgsize, rgsize, myrank;
int *lranks, *rranks;

/* Create the new context ID. Reduce-max to leader within the local
 * group, then find the max between the two leaders, then broadcast
 * within group.
 * In the same message, the leaders exchange their local group sizes
 * and broadcast the received group size to their local group. */

MPI_Group_size(lcomm, &lgsize);
MPI_Comm_rank(lcomm, &myrank);

if (IMPI_max_cid % 2 == 0) IMPI_max_cid += 1;

MPI_Reduce(&IMPI_max_cid, &maxcid, 1, IMPI_UINT8,
           MPI_MAX, lleader, lcomm);

if (lleader == myrank) {
  outmsg[0] = maxcid;
  outmsg[1] = lgsize;

  MPI_Sendrecv(outmsg, 2, MPI_INT, pleader, tag,
               inmsg, 2, MPI_INT, pleader, tag, pcomm, &status);

  if (inmsg[0] < maxcid) inmsg[0] = maxcid;
}

MPI_Bcast(inmsg, 2, IMPI_UINT8, lleader, lcomm);

maxcid = inmsg[0];
rgsize = (int) inmsg[1];

if (maxcid >= IMPI_MAX_CID-1)
  error out of contexts;

newcid = maxcid + 1;
IMPI_max_cid = maxcid + 2;

/* allocate remote group array of ranks */
rranks = malloc(rgsize * sizeof(int));

/* leaders exchange rank arrays and broadcast them to their group */
if (lleader == myrank) {
  lranks = malloc(lgsize * sizeof(int));

  fill local ranks array lranks with the ranks in MPI_COMM_WORLD of
    the process in lcomm;

  MPI_Sendrecv(lranks, lgsize, MPI_INT, pleader, tag, rranks,
```

```
          rgsize, MPI_INT, pleader, tag, pcomm, &status);

    free(lranks);
  }

  MPI_Bcast(rranks, rgsize, MPI_INT, lleader, lcomm);

  /* create the remote group */
  MPI_Comm_group(MPI_COMM_WORLD, &oldgroup);
  MPI_Group_incl(oldgroup, rgsize, rranks, &remotegroup);

  create a new inter-communicator newcomm with point-to-point context
    newcid, collective context (newcid+1), local group the group of
    lcomm and remote group remotegroup;

  return MPI_SUCCESS;
}
```

4.9 Intercomm_merge

```
int MPI_Intercomm_merge(MPI_Comm comm, int high, MPI_Comm *newcomm)
{
  MPI_Status status;
  MPI_Group g1, g2, newgroup;
  IMPI_Uint8 newcid, maxcid;
  IMPI_Uint8 inmsg[2], outmsg[2];
  int myrank, rgsize, rhigh;

  /* Create the new context ID. Rank 0 processes are the leaders of their
   * local group.  Each leader finds the max context ID of all remote
   * group processes (excluding their leader) and their "high" setting.
   * The leaders then swap the information and broadcast to the remote
   * group.
   * Note: this is a criss-cross effect, processes talk to the remote
   * leader. */

  MPI_Comm_rank(comm, &myrank);
  MPI_Comm_remote_size(comm, &rgsize);

  if (IMPI_max_cid % 2 == 0) IMPI_max_cid += 1;

  if (myrank == 0) {
    maxcid = IMPI_max_cid;

    /* find max context ID of remote non-leader processes */
    for (i = 1; i < rgsize; i++) {
      MPI_Recv(inmsg, 1, IMPI_UINT8, i, IMPI_MERGE_TAG, comm, &status);
      if (inmsg[0] > maxcid) maxcid = inmsg[0];
    }

    /* swap context ID and high value with remote leader */
    outmsg[0] = maxcid;
    outmsg[1] = high;

    MPI_Sendrecv(outmsg, 2, IMPI_UINT8, 0, IMPI_MERGE_TAG,
                 inmsg, 2, IMPI_UINT8, 0, IMPI_MERGE_TAG, comm, &status);

    if (inmsg[0] > maxcid) maxcid = inmsg[0];

    rhigh = inmsg[1];

    /* broadcast context ID and local high to remote
     * non-leader processes */
    outmsg[0] = maxcid;
    outmsg[1] = high;

    for (i = 1; i < rgsize; i++)
      MPI_Send(outmsg, 2, IMPI_UINT8, i, IMPI_MERGE_TAG, comm);
  }
  else {
    /* non-leader */
```

```
    MPI_Send(&maxcid, 1, IMPI_UINT8, 0, IMPI_MERGE_TAG, comm);
    MPI_Recv(inmsg, 2, IMPI_UINT8, 0, IMPI_MERGE_TAG, comm, &status);

    maxcid = inmsg[0];
    rhigh = inmsg[1];
  }

  if (maxcid >= IMPI_MAX_CID-1)
    error out of contexts;

  newcid = maxcid + 1;
  IMPI_max_cid = maxcid + 2;

  /* All procs know the "high" for local and remote groups and
   * the context ID. Create the properly ordered union group.
   * In case of equal high values, the group that has the leader
   * with the lowest rank in MPI_COMM_WORLD goes first.
   */
  if (high && (!rhigh)) {
    MPI_Comm_remote_group(comm, &g1);
    MPI_Comm_group(comm, &g2);
  } else if ((!high) && rhigh) {
    MPI_Comm_group(comm, &g1);
    MPI_Comm_remote_group(comm, &g2);
  } else if ((rank in MPI_COMM_WORLD of rank 0 in local group)
         < (rank in MPI_COMM_WORLD of rank 0 in remote group)) {
    MPI_Comm_group(comm, &g1);
    MPI_Comm_remote_group(comm, &g2);
  } else {
    MPI_Comm_remote_group(comm, &g1);
    MPI_Comm_group(comm, &g2);
  }

  MPI_Group_union(g1, g2, &newgroup);

  create a new intra-communicator newcomm with point-to-point context
    newcid, collective context (newcid+1) and group newgroup;

  return MPI_SUCCESS;
}
```

4.10 Barrier

```
int MPI_Barrier(MPI_Comm comm)
{
  MPI_Status status;
  int i, nmasters, myrank, mynum, dim, hibit, mask;

  MPI_Comm_rank(comm, &myrank);

  /* local phase */
  fan in to local master;

  /* global phase */
  if (is_master(myrank, comm)) {
    nmasters = num_masters(comm);
    if (nmasters <= IMPI_MAX_LINEAR_BARRIER) {
      /* linear barrier among the masters */
      if (myrank == 0) {
        for (i = 1; i < nmasters; i++)
          MPI_Recv(MPI_BOTTOM, 0, MPI_BYTE, rank_master(i, comm),
                   IMPI_BARRIER_TAG, comm, &status);

        for (i = 1; i < nmasters; i++)
          MPI_Send(MPI_BOTTOM, 0, MPI_BYTE, rank_master(i, comm),
                   IMPI_BARRIER_TAG, comm);
      } else {
        MPI_Send(MPI_BOTTOM, 0, MPI_BYTE, 0, IMPI_BARRIER_TAG, comm);
        MPI_Recv(MPI_BOTTOM, 0, MPI_BYTE, 0,
                 IMPI_BARRIER_TAG, comm, &status);
      }
    } else {
      /* tree barrier among the masters */
      mynum = master_num(myrank, comm);
      dim = cubedim(nmasters);
      hibit = highbit(mynum, dim);
      --dim;

      /* receive from children */
      for (i = dim, mask = 1 << i; i > hibit; --i, mask >>= 1) {
        peer = mynum | mask;
        if (peer < nmasters)
          MPI_Recv(MPI_BOTTOM, 0, MPI_BYTE, rank_master(peer, comm),
                   IMPI_BARRIER_TAG, comm, &status);
      }

      /* send to and receive from parent */
      if (mynum > 0) {
        peer = rank_master(mynum & ~(1 << hibit), comm);
        MPI_Send(MPI_BOTTOM, 0, MPI_BYTE, peer, IMPI_BARRIER_TAG, comm);

        MPI_Recv(MPI_BOTTOM, 0, MPI_BYTE, peer,
                 IMPI_BARRIER_TAG, comm, &status);
      }
```

```
      /* send to children */
      for (i = hibit + 1, mask = 1 << i; i <= dim; ++i, mask <<= 1) {
        peer = rank | mask;
        if (peer < nmasters)
          MPI_Send(MPI_BOTTOM, 0, MPI_BYTE, rank_master(peer, comm),
                   IMPI_BARRIER_TAG, comm);
      }
    }
  }

  /* local phase */
  fan out from local master;

  return MPI_SUCCESS;
}
```

4.11 Bcast

```
int MPI_Bcast(void *buf, int count, MPI_Datatype dtype,
              int root, MPI_Comm comm)
{
  int myrank;

  MPI_Comm_rank(comm, &myrank);

  /* global phase */
  if (myrank == root ||
      (is_master(myrank, comm) && !are_local(myrank, root, comm)))
    master_bcast(buf, count, dtype, root, comm);

  /* local phase */
  if (are_local(myrank, root, comm))
    broadcast the data from the root to the local processes;
  else
    broadcast the data from the local master to the local processes;

  return MPI_SUCCESS;
}

int master_bcast(void *buf, int count, MPI_Datatype dtype,
                 int root, MPI_Comm comm)
{
  MPI_Status status;
  int myrank, nmasters, mynum, rootnum, vnum, dim, hibit;
  int i, peer, mask;

  MPI_Comm_rank(comm, &myrank);
  nmasters = num_masters(comm);

  if (nmasters <= IMPI_MAX_LINEAR_BCAST) {
    /* linear broadcast between masters */
    if (myrank == root)
```

```
      for (i = 0; i < nmasters; i++) {
        if (i == local_master_num(root, comm)) continue;

        MPI_Send(buf, count, dtype, master_rank(i, comm),
                 IMPI_BCAST_TAG, comm);
      }
    else
      MPI_Recv(buf, count, dtype, root, IMPI_BCAST_TAG, comm, &status);
  } else {
    /* tree broadcast between masters */
    mynum = master_num(myrank, comm);
    rootnum = master_num(root, comm);
    vnum = (mynum + nmasters - rootnum) % nmasters;
    dim = cubedim(nmasters);
    hibit = highbit(vnum, dim);
    --dim;

    /* receive data from parent in the tree */
    if (vnum > 0) {
      peer = ((vnum & ~(1 << hibit)) + rootnum) % nmasters;
      peer = master_rank(peer, comm);
      if (are_local(peer, root, comm))
        peer = root;

      MPI_Recv(buf, count, dtype, peer, IMPI_BCAST_TAG, comm, &status);
    }

    /* send data to the children */
    for (i = hibit + 1, mask = 1 << i; i <= dim; ++i, mask <<= 1) {
      peer = vnum | mask;
      if (peer < nmasters) {
        peer = master_rank((peer + rootnum) % nmasters, comm);
        MPI_Send(buf, count, dtype, peer, IMPI_BCAST_TAG, comm);
      }
    }
  }

  return MPI_SUCCESS;
}
```

4.12 Gather

```
int gather_is_short(int count, MPI_Datatype dtype, MPI_Comm comm)
{
  int size;

  MPI_Pack_size(count, dtype, comm, &size);
  return(size < IMPI_COLL_CROSSOVER);
}

int MPI_Gather(void *sbuf, int scount, MPI_Datatype sdtype, void *rbuf,
```

```
               int rcount, MPI_Datatype rdtype, int root, MPI_Comm comm)
{
  if (gather_is_short(scount, sdtype, comm))
    gather_short(sbuf, scount, sdtype, rbuf, rcount, rdtype, root, comm);
  else
    gather_long(sbuf, scount, sdtype, rbuf, rcount, rdtype, root, comm);

  return MPI_SUCCESS;
}

int gather_long(void *sbuf, int scount, MPI_Datatype sdtype, void *rbuf,
                int rcount, MPI_Datatype rdtype, int root, MPI_Comm comm)
{
  MPI_Status status;
  MPI_Aint extent;
  int i, nprocs, myrank, incr;
  char *p;

  MPI_Comm_rank(comm, &myrank);
  MPI_Comm_size(comm, &nprocs);

  if (myrank != root) {
    MPI_Send(sbuf, scount, sdtype, root, IMPI_GATHER_TAG, comm);
    return MPI_SUCCESS;
  }

  MPI_Type_extent(rdtype, &extent);
  incr = extent * rcount;

  for (i = 0, p = (char *) rbuf; i < nprocs; i++, p += incr) {
    if (i == myrank)
      MPI_Sendrecv(sbuf, scount, sdtype, i, IMPI_GATHER_TAG,
                   p, rcount, rdtype, i, IMPI_GATHER_TAG, comm, &status);
    else
      MPI_Recv(p, rcount, rdtype, i, IMPI_GATHER_TAG, comm, &status);
  }

  return MPI_SUCCESS;
}

int gather_short(void *sbuf, int scount, MPI_Datatype sdtype, void *rbuf,
                 int rcount, MPI_Datatype rdtype, int root, MPI_Comm comm)
{
  MPI_Status status;
  int myrank, packsize, vnum, rootnum, nmasters;
  int mask, nprocs, count, size;
  int mynum, peer, i;
  char *tmpbuf;

  MPI_Comm_rank(comm, &myrank);
  MPI_Comm_size(comm, &nprocs);
  MPI_Pack_size(scount, sdtype, comm, &packsize);

```

```
if (is_master(myrank, comm) || myrank == root)
  allocate a temporary buffer tmpbuf of size nprocs*packsize;

nmasters = num_masters(comm);

/* local phase */
if (are_local(myrank, root, comm))
  gather the send buffers of the local processes into the
    root's receive buffer;
else
  gather send buffers at the local master into tmpbuf;
  /* At this point the master must have a buffer tmpbuf
   * containing a concatenation in rank order of the
   * local processes packed send buffers.
   */

/* global phase */
if ((myrank == root) || (is_master(myrank, comm)
    && !are_local(myrank, root, comm))) {

  if (nmasters <= IMPI_MAX_LINEAR_GATHER) {
    /* linear gather to root */
    if (myrank == root) {
      for (i = 0, size = 0; i < nmasters; i++) {
        if (i == local_master_num(root, comm))
          continue;            /* skip root's node */

        MPI_Recv(tmpbuf+size, nprocs*packsize, MPI_BYTE,
                 master_rank(i, comm), IMPI_GATHER_TAG, comm, &status);
        MPI_Get_count(status, MPI_BYTE, &count);
        size += count;
      }
    } else {
      size = num_local_to_rank(myrank, comm) * packsize;
      MPI_Send(tmpbuf, size, MPI_BYTE, root, IMPI_GATHER_TAG, comm);
    }
  } else {
    /* tree gather to root */
    mynum = local_master_num(myrank, comm);
    rootnum = local_master_num(root, comm);
    vnum = (mynum - rootnum + nmasters) % nmasters;

    if (myrank == root)
        size = 0;
    else
      size = num_local_to_rank(myrank, comm) * packsize;

    for (mask = 1; mask < nprocs; mask <<= 1) {
      if (vnum & mask) {
        peer = master_rank(((vnum & ~mask) + rootnum) % nmasters, comm);
        if (are_local(peer, root, comm))
          peer = root;
```

```
          MPI_Send(tmpbuf, size, MPI_BYTE, peer, IMPI_GATHER_TAG, comm);
          break;
        }
        else {
          peer = vnum | mask;
          if (peer >= nmasters) continue;
          peer = master_rank((peer + rootnum) % nmasters, comm);
          if (are_local(peer, root, comm))
            peer = root;

          MPI_Recv(tmpbuf+size, nprocs*packsize, MPI_BYTE, peer,
                   IMPI_GATHER_TAG, comm, &status);
          MPI_Get_count(status, MPI_BYTE, &count);
          size += count;
        }
      }
    }
  }

  /* local phase */
  if (myrank == root) {
    /* For the linear gather to root, tmpbuf contains, concatenated in
     * order of master rank, the concatenations of the process send
     * buffers created in the first local phase.
     * For the tree gather to root, the order of these send buffers can be
     * circularly rotated by master rank number (skipping over the root,
     * which has been put directly in the root's receive buffer already).
     */

    unpack the data in tmpbuf into the receive buffer;
  }

  if (is_master(myrank, comm))
    free(tmpbuf);

  return MPI_SUCCESS;
}
```

4.13 Gatherv

```
IMPI_Int4 gatherv_is_short(int *count, MPI_Datatype dtype,
                           int root, MPI_Comm comm)
{
  IMPI_Int4   maxsize;
  int         myrank, nprocs, size;

  MPI_Comm_rank(comm, &myrank);
  MPI_Comm_size(comm, &nprocs);
  MPI_Pack_size(1, dtype, comm, &size);

  if (myrank == root) {
    maxsize = count[0] * size;
```

```
    for (i = 1; i < nprocs; i++)
      if (count[i] * size > maxsize)
        maxsize = count[i] * size;

    if (maxsize > IMPI_COLL_CROSSOVER)
      maxsize = 0;
  }

  MPI_Bcast(&maxsize, 1, IMPI_INT4, root, comm);
  return(maxsize);
}

int MPI_Gatherv(void *sbuf, int scount, MPI_Datatype sdtype,
                void *rbuf, int *rcounts, int *disps, MPI_Datatype rdtype,
                int root, MPI_Comm comm)
{
  IMPI_Int4 maxsize;

  maxsize = gatherv_is_short(rcounts, rdtype, root, comm);
  if (maxsize)
    gatherv_short(sbuf, scount, sdtype, rbuf, rcounts, disps, rdtype,
                  root, comm, maxsize);
  else
    gatherv_long(sbuf, scount, sdtype, rbuf, rcounts, disps, rdtype,
                 root, comm);

  return MPI_SUCCESS;
}

int gatherv_short(void *sbuf, int scount, MPI_Datatype sdtype,
                  void *rbuf, int *rcounts, int *disps,
                  MPI_Datatype rdtype,
                  int root, MPI_Comm comm, IMPI_Int4 maxsize)
{
  MPI_Status status;
  int myrank, packsize, vnum, rootnum, nmasters;
  int mask, nprocs, count, size;
  int i, msgnum, peer;
  char *tmpbuf;

  MPI_Comm_rank(comm, &myrank);
  MPI_Comm_size(comm, &nprocs);

  if (is_master(myrank, comm) || myrank == root)
    allocate a temporary buffer tmpbuf of size nprocs*maxsize;

  nmasters = num_masters(comm);

  /* local phase */
  if (are_local(myrank, root, comm)) {
    gather the send buffers of the local processes into the
      root's receive buffer;
  } else {
```

```
    gather send buffers at the local master into tmpbuf;
    on local master set size equal to the # of bytes gathered in tmpbuf;
    /* At this point the master must have a buffer tmpbuf
     * containing a concatenation in rank order of the
     * local processes packed send buffers.
     */
  }

  /* global phase */
  if ((myrank == root) || (is_master(myrank, comm)
      && !are_local(myrank, root, comm))) {

    if (nmasters <= IMPI_MAX_LINEAR_GATHER) {
      /* linear gather to root */
      if (myrank == root) {
        for (i = 0, size = 0; i < nmasters; i++) {
          if (i == local_master_num(root, comm))
            continue;            /* skip root's node */

          MPI_Recv(tmpbuf+size, nprocs*maxsize, MPI_BYTE,
                   master_rank(i, comm), IMPI_GATHERV_TAG, comm, &status);
          MPI_Get_count(status, MPI_BYTE, &count);
          size += count;
        }
      } else
        MPI_Send(tmpbuf, size, MPI_BYTE, root, IMPI_GATHERV_TAG, comm);
    } else {
      /* tree gather to root */
      mynum = local_master_num(myrank, comm);
      rootnum = local_master_num(root, comm);
      vnum = (mynum - rootnum + nmasters) % nmasters;

      if (myrank == root)
          size = 0;

      for (mask = 1; mask < nprocs; mask <<= 1) {
        if (vnum & mask) {
          peer = master_rank(((vnum & ~mask) + rootnum) % nmasters, comm);
          if (are_local(peer, root, comm))
            peer = root;

          MPI_Send(tmpbuf, size, MPI_BYTE, peer, IMPI_GATHERV_TAG, comm);
          break;
        }
        else {
          peer = vnum | mask;
          if (peer >= nmasters) continue;
          peer = master_rank((peer + rootnum) % nmasters, comm);
          if (are_local(peer, root, comm))
            peer = root;

          MPI_Recv(tmpbuf+size, nprocs*maxsize, MPI_BYTE, peer,
                   IMPI_GATHERV_TAG, comm, &status);
```

```
          MPI_Get_count(status, MPI_BYTE, &count);
          size += count;
        }
      }
    }
  }

  /* local phase */
  if (myrank == root) {
    /* tmpbuf contains concatenated in order of master rank the
     * concatenations of the process send buffers created in the first
     * local phase
     */
    unpack the data in tmpbuf into the receive buffer;
  }

  if (is_master(myrank, comm))
    free(tmpbuf);

  return MPI_SUCCESS;
}

int gatherv_long(void *sbuf, int scount, MPI_Datatype sdtype,
                 void *rbuf, int *rcounts, int *disps,
                 MPI_Datatype rdtype, int root, MPI_Comm comm)
{
  MPI_Status status;
  MPI_Aint extent;
  int i, myrank, nprocs;
  char *p;

  MPI_Comm_rank(comm, &myrank);
  MPI_Comm_size(comm, &nprocs);

  if (myrank != root) {
    MPI_Send(sbuf, scount, sdtype, root, IMPI_GATHER_TAG, comm);
    return MPI_SUCCESS;
  }
  MPI_Type_extent(rdtype, &extent);

  for (i = 0; i < nprocs; i++) {

    p = ((char *) rbuf) + (extent * disps[i]);

    if (i == myrank)
      MPI_Sendrecv(sbuf, scount, sdtype, i, IMPI_GATHERV_TAG,
                   p, rcounts[i], rdtype, i, IMPI_GATHERV_TAG,
                   comm, &status);
    else
      MPI_Recv(p, rcounts[i], rdtype, i, IMPI_GATHERV_TAG, comm, &status);
  }
  return MPI_SUCCESS;
}
```

4.14 Scatter

```
int scatter_is_short(int count, MPI_Datatype dtype, MPI_Comm comm)
{
  int size;

  MPI_Pack_size(count, dtype, comm, &size);
  return(size < IMPI_COLL_CROSSOVER);
}

int MPI_Scatter(void *sbuf, int scount, MPI_Datatype sdtype,
                void *rbuf, int rcount, MPI_Datatype rdtype,
                int root, MPI_Comm comm)
{
  if (scatter_is_short(rcount, rdtype, comm))
    scatter_short(sbuf, scount, sdtype, rbuf, rcount, rdtype, root, comm);
  else
    scatter_long(sbuf, scount, sdtype, rbuf, rcount, rdtype, root, comm);

  return MPI_SUCCESS;
}

int scatter_long(void *sbuf, int scount, MPI_Datatype sdtype,
                 void *rbuf, int rcount, MPI_Datatype rdtype,
                 int root, MPI_Comm comm)
{
  MPI_Status status;
  MPI_Aint extent;
  int i, myrank, nprocs, incr;
  char *p;

  MPI_Comm_rank(comm, &myrank);
  MPI_Comm_size(comm, &nprocs);

  if (myrank != root)
    MPI_Recv(rbuf, rcount, rdtype, root, IMPI_SCATTER_TAG, comm, &status);
  else {
    MPI_Type_extent(sdtype, &extent);
    incr = extent * scount;

    for (i = 0, p = (char *) sbuf; i < nprocs; i++, p += incr) {
      if (i == myrank)
        MPI_Sendrecv(p, scount, sdtype, i, IMPI_SCATTER_TAG,
                     rbuf, rcount, rdtype, i, IMPI_SCATTER_TAG,
                     comm, &status);
      else
        MPI_Send(p, scount, sdtype, i, IMPI_SCATTER_TAG, comm);
    }
  }

  return MPI_SUCCESS;
}
```

```
int scatter_short(void *sbuf, int scount, MPI_Datatype sdtype,
                  void *rbuf, int rcount, MPI_Datatype rdtype,
                  int root, MPI_Comm comm)
{
  MPI_Status status;
  int i, myrank, packsize, size, nmasters;
  void *tmpbuf;

  MPI_Comm_rank(comm, &myrank);
  MPI_Pack_size(rcount, rdtype, comm, &packsize);

  /* global phase */
  if (myrank == root) {
    nmasters = num_masters(comm);
    for (i = 0; i < nmasters; i++) {
      if (i == local_master_num(root, comm))
        continue;        /* skip root's node */

      size = num_local_to_master(i, comm) * packsize;

      allocate a temporary buffer tmpbuf of size bytes and
        put into it concatenated in rank order packed copies of the data
        destined for each process local to master i;

      MPI_Send(tmpbuf, size, MPI_BYTE,
               master_rank(i, comm), IMPI_SCATTER_TAG, comm);

      free tmpbuf;
    }
  } else if (is_master(myrank, comm) && !are_local(myrank, root, comm)) {
    size = num_local_to_rank(myrank, comm) * packsize;
    allocate a temporary buffer tmpbuf of size bytes;
    MPI_Recv(tmpbuf, size, MPI_BYTE, root,
             IMPI_SCATTER_TAG, comm, &status);
  }

  /* local phase */
  if (are_local(myrank, root, comm))
    scatter data from root sbuf to local processes;
  else
    scatter packed data from master tmpbuf to local processes;

  free all temporary buffers which are still allocated;
  return MPI_SUCCESS;
}
```

4.15 Scatterv

```
IMPI_Int4 scatterv_is_short(int *count, MPI_Datatype dtype,
                            int root, MPI_Comm comm)
```

```
{
  IMPI_Int4   maxsize;
  int         i, myrank, nprocs, size;

  MPI_Comm_rank(comm, &myrank);
  MPI_Comm_size(comm, &nprocs);
  MPI_Pack_size(1, dtype, comm, &size);

  if (myrank == root) {
    maxsize = count[0] * size;
    for (i = 1; i < nprocs; i++)
      if (count[i] * size > maxsize)
        maxsize = count[i] * size;

    if (maxsize > IMPI_COLL_CROSSOVER)
      maxsize = 0;
  }

  MPI_Bcast(&maxsize, 1, IMPI_INT4, root, comm);
  return(maxsize);
}

int MPI_Scatterv(void *sbuf, int *scounts, int *disps,
                 MPI_Datatype sdtype,
                 void *rbuf, int rcount, MPI_Datatype rdtype,
                 int root, MPI_Comm comm)
{
  IMPI_Int4 maxsize;

  maxsize = scatterv_is_short(scounts, sdtype, root, comm);
  if (maxsize)
    scatterv_short(sbuf, scounts, disps, sdtype, rbuf, rcount, rdtype,
                   root, comm, maxsize);
  else
    scatterv_long(sbuf, scounts, disps, sdtype, rbuf, rcount, rdtype,
                  root, comm);

  return MPI_SUCCESS;
}


/* find sum of the counts to be scattered to the processes
 * local to master number i
 */
int sum_counts_to_master(int *counts, int m, MPI_Comm comm)
{
  int *ranks;
  int i, nranks, sum;

  nranks = num_local_to_master(m, comm);
  ranks = locals_to_master(m, comm);

  for (i = 0, sum = 0; i < nranks; i++)
```

```
    sum += counts[ranks[i]];

  free(ranks);
  return(sum);
}

int scatterv_short(void *sbuf, int *scounts, int *disps,
                   MPI_Datatype sdtype,
                   void *rbuf, int rcount, MPI_Datatype rdtype,
                   int root, MPI_Comm comm, IMPI_Int4 maxsize)
{
  MPI_Status status;
  int i, myrank, packsize, size, nmasters;
  void *tmpbuf;

  MPI_Comm_rank(comm, &myrank);
  MPI_Pack_size(1, sdtype, comm, &packsize);

  /* global phase */
  if (myrank == root) {
    nmasters = num_masters(comm);
    for (i = 0; i < nmasters; i++) {
      if (i == local_master_num(root, comm))
        continue;        /* skip root's node */

      size = sum_counts_to_master(scounts, i, comm) * packsize;

      create a temporary buffer tmpbuf of size bytes and
        put into it concatenated in rank order packed copies of the data
        destined for each process local to master i;

      MPI_Send(tmpbuf, size, MPI_BYTE,
               master_rank(i, comm), IMPI_SCATTERV_TAG, comm);

      free tmpbuf;
    }
  }
  else if (is_master(myrank, comm) && !are_local(myrank, root, comm)) {
    size = num_local_to_rank(myrank, comm) * maxsize;
    allocate a temporary buffer tmpbuf of size bytes;
    MPI_Recv(tmpbuf, size, MPI_BYTE, root,
             IMPI_SCATTERV_TAG, comm, &status);
  }

  /* local phase */
  if (are_local(myrank, root, comm))
    scatter data from root sbuf to local processes;
  else
    scatter packed data from master tmpbuf to local processes;

  free all temporary buffers which are still allocated;
  return MPI_SUCCESS;
}
```

```
int scatterv_long(void *sbuf, int *scounts, int *disps,
                  MPI_Datatype sdtype,
                  void *rbuf, int rcount, MPI_Datatype rdtype,
                  int root, MPI_Comm comm)
{
  MPI_Status  status;
  MPI_Aint    extent;
  int         i, myrank, nprocs;
  char        *p;

  MPI_Comm_rank(comm, &myrank);
  MPI_Comm_size(comm, &nprocs);

  if (myrank != root) {
    MPI_Recv(rbuf, rcount, rdtype, root, IMPI_SCATTERV_TAG,
             comm, &status);
    return MPI_SUCCESS;
  }

  MPI_Type_extent(sdtype, &extent);

  for (i = 0; i < nprocs; i++) {
    p = ((char *) sbuf) + (extent * disps[i]);
    if (i == myrank)
      MPI_Sendrecv(p, scounts[i], sdtype, i, IMPI_SCATTERV_TAG,
                   rbuf, rcount, rdtype, i, IMPI_SCATTERV_TAG,
                   comm, &status);
    else
      MPI_Send(p, scounts[i], sdtype, i, IMPI_SCATTERV_TAG, comm);
  }

  return MPI_SUCCESS;
}
```

4.16 Reduce

```
int MPI_Reduce(void *sbuf, void *rbuf, int count, MPI_Datatype dtype,
               MPI_op op, int root, MPI_Comm comm)
{
 if (op is commutative)
  return(reduce_commutative(sbuf, rbuf, count, dtype, op, root, comm));
 else
  return(reduce_noncommutative(sbuf, rbuf, count, dtype, op, root, comm));
}

int reduce_commutative(void *sbuf, void *rbuf, int count,
                       MPI_Datatype dtype,
                       MPI_op op, int root, MPI_Comm comm)
{
  MPI_Status status;
  int myrank, mynum, rootnum, vnum, dim, hibit, mask;
  int peer, nmasters, i;
```

```
void *tmpbuf, *redbuf;

nmasters = num_masters(comm);
MPI_Comm_rank(comm, &myrank);

/* local phase */
if (are_local(myrank, root, comm))
  perform a local reduction to root's rbuf;
else
  perform a local reduction to a temporary buffer redbuf in
    the local master;

/* global phase */
if ((myrank == root) || (is_master(myrank, comm)
    && !are_local(myrank, root, comm))) {

  allocate a temporary buffer tmpbuf large enough for
    count copies of dtype;

  if (nmasters <= IMPI_MAX_LINEAR_REDUCE) {
    /* linear reduction to root */
    if (myrank == root) {
      for (i = 0; i < nmasters; i++) {
        if (i == local_master_num(root, comm)) continue;
        MPI_Recv(tmpbuf, count, dtype, master_rank(i, comm),
                 IMPI_REDUCE_TAG, comm, &status);
      }

      call reduction function op on tmpbuf (invec)
        and rbuf (inoutvec);
    } else {
      MPI_Send(redbuf, count, dtype, root, IMPI_REDUCE_TAG, comm);
    }
  } else {
    /* tree reduction to root */
    mynum = local_master_num(myrank, comm);
    rootnum = local_master_num(root, comm);
    vnum = (mynum - rootnum + nmasters) % nmasters;
    dim = cubedim(nmasters);

    /* loop over cube dimensions */
    for (i = 0, mask = 1; i < dim; ++i, mask <<= 1) {
      /* a high-proc sends to low-proc and stops */
      if (vnum & mask) {
        peer = master_rank(((vnum & ~mask) + rootnum) % nmasters, comm);
        if (are_local(peer, root, comm))
          peer = root;

        MPI_Send(redbuf, count, dtype, peer, IMPI_REDUCE_TAG, comm);
        break;
      }

      /* a low-proc receives, reduces, and moves
```

```
         * to a higher dimension */
        else {
          peer = vnum | mask;
          if (peer >= nmasters) continue;
          peer = master_rank((peer + rootnum) % nmasters, comm);

          MPI_Recv(tmpbuf, count, dtype, peer,
                   IMPI_REDUCE_TAG, comm, &status);

          if (myrank == root) {
            call reduction function op on tmpbuf (invec)
              and rbuf (inoutvec);
          } else {
            call reduction function op on tmpbuf (invec)
              and redbuf (inoutvec);
          }
        }
      }
    }
  }

  free all temporary buffers;
  return MPI_SUCCESS;
}

int reduce_noncommutative(void *sbuf, void *rbuf, int count,
                MPI_Datatype dtype, MPI_op op, int root, MPI_Comm comm)
{
  MPI_Status status;
  int i, myrank, nprocs;
  void *inbuf, *tmpbuf;

  MPI_Comm_size(comm, &nprocs);
  MPI_Comm_root(comm, &myrank);

  if (myrank != root)
    return(MPI_Send(sbuf, count, dtype, root, IMPI_REDUCE_TAG, comm);

  if (nprocs > 1)
    create a temporary buffer tmpbuf large enough for count dtypes;

  if (myrank == (nprocs - 1))
    MPI_Sendrecv(sbuf, count, dtype, myrank, IMPI_REDUCE_TAG,
                 rbuf, count, dtype, myrank, IMPI_REDUCE_TAG, comm,
                 &status);
  else
    MPI_Recv(rbuf, count, dtype, nprocs - 1, IMPI_REDUCE_TAG, comm,
             &status);

  for (i = nprocs - 2; i >= 0; --i) {
    if (myrank == i) {
       inbuf = sbuf;
    } else {
```

```
      MPI_Recv(tmpbuf, count, dtype, i, IMPI_REDUCE_TAG, comm, &status);
      inbuf = tmpbuf;
    }

    call reduction function op on inbuf (invec) and rbuf (inoutvec);
  }

  free all temporary buffers;
  return MPI_SUCCESS;
}
```

4.17 Reduce_scatter

```
int MPI_Reduce_scatter(void *sbuf, void *rbuf, int *rcounts,
                       MPI_Datatype dtype, MPI_Op op, MPI_Comm comm)
{
  void *tmpbuf = 0;
  int *disps = 0;
  int i, myrank, nprocs, count;

  MPI_Comm_rank(comm, &myrank);
  MPI_Comm_size(comm, &nprocs);

  if (myrank == 0) {
    for (i = 0, count = 0; i < nprocs; i++) {
      count += rcounts[i];
    }
    create a temporary buffer tmpbuf large enough for count dtypes;
    disps = malloc(nprocs * sizeof(int));

    disps[0] = 0;
    for (i = 0; i < (nprocs - 1); i++)
      disps[i + 1] = disps[i] + rcounts[i];
  }

  MPI_Reduce(sbuf, tmpbuf, count, dtype, op, 0, comm);

  MPI_Scatterv(tmpbuf, rcounts, disps, dtype,
               rbuf, rcounts[myrank], dtype, 0, comm);

  if (disps) free(disps);
  if (tmpbuf) free(tmpbuf);
  return MPI_SUCCESS;
}
```

4.18 Scan

```
int MPI_Scan(void *sbuf, void *rbuf, int count,
             MPI_Datatype dtype, MPI_Op op, MPI_Comm comm)
{
  MPI_Status status;
```

```
  int i, myrank, nprocs;
  void *tmpbuf;

  MPI_Comm_rank(comm, &myrank);
  MPI_Comm_size(comm, &nprocs);

  /* copy the send buffer into the receive buffer */
  MPI_Sendrecv(sbuf, count, dtype, myrank, IMPI_SCAN_TAG,
               rbuf, count, dtype, myrank, IMPI_SCAN_TAG, comm, &status);

  if (myrank > 0) {
    /* receive previous buffer into a temporary and reduce */

    create a temporary buffer tmpbuf large enough for count dtypes;

    MPI_Recv(tmpbuf, count, dtype, myrank-1, IMPI_SCAN_TAG, comm,
             &status);

    call reduction function op on tmpbuf (invec) and rbuf (inoutvec)

    free tmpbuf;
  }

  if (myrank < (nprocs - 1))
      /* send result to next process */
      MPI_Send(rbuf, count, dtype, myrank + 1, IMPI_SCAN_TAG, comm);

  return MPI_SUCCESS;
}
```

4.19 Allgather

```
int MPI_Allgather(void *sbuf, int scount, MPI_Datatype sdtype,
                  void *rbuf, int rcount, MPI_Datatype rdtype,
                  MPI_Comm comm)
{
  int nprocs;

  MPI_Comm_size(comm, &nprocs);
  MPI_Gather(sbuf, scount, sdtype, rbuf, rcount, rdtype, 0, comm);
  MPI_Bcast(rbuf, rcount * nprocs, rdtype, 0, comm);
  return MPI_SUCCESS;
}
```

4.20 Allgatherv

```
int
MPI_Allgatherv(void *sbuf, int scount, MPI_Datatype sdtype,
               void *rbuf, int *rcounts,
               int *displs, MPI_Datatype rdtype,
               MPI_Comm comm)
```

```
{
 int nprocs;
 int i, total_rcount;

 MPI_Comm_size(comm, &nprocs);
 MPI_Gatherv(sbuf, scount, sdtype, rbuf, rcounts, displs, rdtype, 0,comm);
 total_rcount = 0;
 for (i=0, total_rcount=0; i<nprocs; i++)
   total_rcount += rcounts[i];

 MPI_Bcast(rbuf, total_rcount, rdtype, 0, comm);
 return MPI_SUCCESS;
}
```

4.21 Allreduce

```
int MPI_Allreduce(void *sbuf, void *rbuf, int count, MPI_Datatype dtype,
                  MPI_op op, MPI_Comm comm)
{
  MPI_Reduce(sbuf, rbuf, count, dtype, op, 0, comm);
  MPI_Bcast(rbuf, count, dtype, 0, comm);
  return MPI_SUCCESS;
}
```

4.22 Alltoall

```
int alltoall_is_short(int count, MPI_Datatype dtype, MPI_Comm comm)
{
  int size;

  MPI_Pack_size(1, dtype, comm, &size);
  return(count * size < IMPI_COLL_CROSSOVER);
}

int MPI_Alltoall(void *sbuf, int scount, MPI_Datatype sdtype,
                void *rbuf, int rcount, MPI_Datatype rdtype,
                MPI_Comm comm)
{
  if (alltoall_is_short(scount, sdtype, comm))
   alltoall_short(sbuf, scount, sdtype, rbuf, rcount, rdtype, root, comm);
  else
   alltoall_long(sbuf, scount, sdtype, rbuf, rcount, rdtype, root, comm);
}

int alltoall_long(void *sbuf, int scount, MPI_Datatype sdtype,
                  void *rbuf, int rcount, MPI_Datatype rdtype,
                  MPI_Comm comm)
{
  MPI_Request *reqs;
  MPI_Status  *stats;
  MPI_Aint    sextent, rextent;
```

```
  int         i, nprocs;

  MPI_Comm_size(comm, &nprocs);
  MPI_Type_extent(sdtype, &sextent);
  MPI_Type_extent(rdtype, &rextent);

  reqs = (MPI_Request *) malloc(2 * nprocs * sizeof(MPI_Request));
  stats = (MPI_Status *) malloc(2 * nprocs * sizeof(MPI_Status));

  for (i = 0; i < nprocs; i++) {
    MPI_Irecv((char *) rbuf + i * rcount * rextent, rcount,
              rdtype, i, IMPI_ALLTOALL_TAG, comm, &reqs[2*i]);
    MPI_Isend((char *) sbuf + i * scount * sextent, scount,
              sdtype, i, IMPI_ALLTOALL_TAG, comm, &reqs[2*i + 1]);
  }

  MPI_Waitall(2 * nprocs, reqs, stats);

  free(reqs);
  free(stats);
  return MPI_SUCCESS;
}

int alltoall_short(void *sbuf, int scount, MPI_Datatype sdtype,
                   void *rbuf, int rcount, MPI_Datatype rdtype,
                   MPI_Comm comm)
{
  MPI_Status  status;
  int         i, j, myrank, nmasters, packsize, size;
  int         rootmaster, nroot;

  MPI_Comm_rank(comm, &myrank);
  MPI_Pack_size(scount, sdtype, comm, &packsize);

  /* local phase */
  do local all to all exchange;

  /* global phase */
  /* This phase rotates around the nodes treating each one in turn as
   * the root. The root node master collects in turn the buffers
   * destined for each other node and sends them in a single transfer
   * to the node where a local operation scatters them to the local
   * destination processes.
   */
  nmasters = num_masters(comm);
  for (i = 0; i < nmasters; i++) {
    rootmaster = master_rank(i, comm);
    nroot = num_local_to_master(i, comm);

    if (are_local(myrank, rootmaster, comm)) {
      for (j = 0; j < nmasters; j++) {
        if (i == j) continue;
```

```
        if (myrank == rootmaster) {
          size = nroot * num_local_to_master(j, comm) * packsize;
          allocate a temporary buffer tmpbuf of size bytes;
        }

        gather into the tmpbuf at rootmaster all send buffers
          destined from processes on node i to processes on node j, they
          are concatenated in order of sender rank by receiver rank;

        if (myrank == rootmaster) {
          MPI_Send(tmpbuf, size, MPI_BYTE, master_rank(j, comm),
                   MPI_ALLTOALL_TAG, comm);
          free tmpbuf;
        }
      }
    } else {
      /* not local to the rootmaster */
      if (is_master(myrank, comm)) {
          size = nroot * num_local_to_rank(myrank, comm) * packsize;
          allocate a temporary buffer tmpbuf of size bytes;

          MPI_Recv(tmpbuf, size, MPI_BYTE, rootmaster,
                   MPI_ALLTOALL_TAG, comm, &status);
      }

      scatter the packed send buffers received from rootmaster from
        the tmpbuf of the local master to the local processes;

      if (is_master(myrank, comm))
        free tmpbuf;
    }
  }

  return MPI_SUCCESS;
}
```

4.23 Alltoallv

```
int MPI_Alltoallv(void *sbuf, int *scounts, int *sdisps,
                  MPI_Datatype sdtype,
                  void *rbuf, int *rcounts, int *rdisps,
                  MPI_Datatype rdtype,
                  MPI_Comm comm)
{
  MPI_Request *reqs;
  MPI_Status  *stats;
  MPI_Aint    sextent, rextent;
  int         i, nprocs;

  MPI_Comm_size(comm, &nprocs);
  MPI_Type_extent(sdtype, &sextent);
  MPI_Type_extent(rdtype, &rextent);
```

```
  reqs = (MPI_Request *) malloc(2 * nprocs * sizeof(MPI_Request));
  stats = (MPI_Status *) malloc(2 * nprocs * sizeof(MPI_Status));

  for (i = 0; i < nprocs; i++) {
    MPI_Irecv((char *) rbuf + rdisps[i] * rextent, rcounts[i],
              rdtype, i, IMPI_ALLTOALLV_TAG, comm, &reqs[2*i]);
    MPI_Isend((char *) sbuf + sdisps[i] * sextent, scounts[i],
              sdtype, i, IMPI_ALLTOALLV_TAG, comm, &reqs[2*i + 1]);
  }

  MPI_Waitall(2 * nprocs, reqs, stats);

  free(reqs);
  free(stats);
  return MPI_SUCCESS;
}
```

4.24 Finalize

```
int MPI_Finalize(void)
{
  MPI_Barrier(MPI_COMM_WORLD);
  send finalize message to host;
  do implementation dependent clean up;
  return MPI_SUCCESS;
}
```

4.25 Constants

```
IMPI_UINT8_MAX          18446744073709551615
IMPI_MAX_CID            18446744073709551600

IMPI_BARRIER_TAG        110
IMPI_BCAST_TAG          120
IMPI_GATHER_TAG         130
IMPI_GATHERV_TAG        140
IMPI_SCATTER_TAG        150
IMPI_SCATTERV_TAG       160
IMPI_ALLTOALL_TAG       170
IMPI_ALLTOALLV_TAG      180
IMPI_REDUCE_TAG         190
IMPI_SCAN_TAG           200
IMPI_DUP_TAG            210
IMPI_MERGE_TAG          220
IMPI_COMM_FREE_TAG      230

IMPI_COLL_CROSSOVER     1024

IMPI_MAX_LINEAR_REDUCE  4
IMPI_MAX_LINEAR_GATHER  4
IMPI_MAX_LINEAR_BCAST   4
IMPI_MAX_LINEAR_BARRIER 4
```

4.26 Future work

The collectives are defined so that for each client there is one process, the master, which participates in the global communication phases. In cases where there are multiple hosts per client it is reasonable to expect that better performance may be obtained by having multiple processes, one per host, participating in the global communication phases. The current collectives can easily be extended to this model by changing the definition of master process so that there is one per host rather than one per client. In addition, to allow maximal exploitation of native communication, it may be necessary to modify some of the collectives so that local phases remain defined between all processes in a client rather than between all processes local to a host.

Chapter 5

IMPI Conformance Testing

5.1 Summary

This chapter describes a Web-based IMPI conformance testing system. This testing system is intended to assist implementors of IMPI and to verify compliance of their implementation to the protocol defined in this document. Should a dispute occur over what the IMPI protocol specifies, the IMPI document, *not* this tester, should be considered the final word. This is a work in progress, comments and suggestions are welcome.

This tester is designed to verify the correct implementation of the IMPI specific protocols, not to test an MPI implementation against the MPI specifications. The full testing of MPI is well beyond the scope of this tester. The IMPI tester operates only within the C implementation of MPI. The testing of IMPI within a Fortran or C++ environment is not yet planned.

To help describe this testing environment, some conformance testing terminology will be used. The implementation of the IMPI protocol to be tested will be referred to as the *Implementation Under Test* (IUT). The IUT will be running on a *System Under Test* (SUT). The SUT refers to all the hardware and software needed to run the IUT. The person running the test will be called the *tester*.

The steps taken by a tester to run the IMPI conformance tests are outlined below. Figure 5.1 shows this testing scheme graphically. The numbers in this figure indicate the order in which the communications channels are established initially. The dashed lines are HTTP Web communication (connection-less communications, stateless). The solid lines are connection-oriented TCP/IP sockets. The dotted line is direct tester interaction with the SUT.

- The *Tester* connects to the NIST IMPI web page at `http://impi.nist.gov/IMPI`. (Figure 5.2) with a Java-enabled browser. The current version of the IMPI standard (this document) is available from this web page.

- Follow the **IMPI Test Tool** link to the main IMPI Test Tool page. This page gives a short description of the major parts of the IMPI testing software: the Test Interpreter, the Test Tool Applet, the Test Scripts, and the Test Server.

- Follow the **Test Interpreter** link to obtain the current version of the test interpreter. The user must compile and link this interpreter for the *IUT* on the *SUT* before continuing with the tests.

- Follow the **Test Scripts** link to view the source to the test scripts and descriptions of the available tests.

- Follow the **Test Tool Applet** link when you are ready to begin testing. This page gives detailed instructions on running the tests.

- Selecting the **Run the IMPI Test Tool Applet** link will initiate the IMPI Test Tool (a java client applet) which will be down-loaded and run on the tester's browser (see Figure 5.4). This applet establishes a permanent[1] TCP/IP connection with an instance of the IMPI Test Manager (the test server) on the NIST IMPI Web server. Test requests and results will be exchanged over this socket.

- The tester can choose the configuration of clients, hosts, and processes to be tested through the Test Tool. This includes choosing where the `impirun -server` process will be run, how many clients there will be, how many of these clients will be on the NIST side (simulated clients), how many hosts will be on each simulated client, how many processes will be on each of these simulated hosts, and what rank to assign to the first simulated client.

  This does not allow some valid configurations to be specified, such as varying the number of hosts per simulated client. If this becomes necessary for the proper testing of the IMPI protocol, then this interface will be modified to allow more general configurations.

- Once the configuration of clients and hosts is specified, the IMPI Test Tool allows the tester to run the IMPI Startup protocol only, for early testing of an implementation. The **Run Startup Only** button on the Test Tool initiates this test. This option runs through the startup on each client with each process printing its rank to `stdout` for confirmation of the ordering within `MPI_COMM_WORLD`. Each process exits before starting the test interpreter. When running startup-only, the NIST side sends a trace of the progress of the startup protocol to the Test Tool so that errors in the Startup processing can be more easily identified. This tracing does not occur otherwise.

  The MPI routines exercised under this option are `MPI_Init`, `MPI_Comm_size`, `MPI_Comm_rank`, and `MPI_Finalize`. Unfortunately, `MPI_Finalize` is not a local operation in IMPI since it executes an `MPI_Barrier`. So for startup-only to complete cleanly, the SUT must have this collective operation already implemented. This should still be useful to the tester early in the implementation, even if the tester hangs in the `MPI_Finalize` call each time.

  Errors that occur during the startup communications will be reported to the tester through the Test Tool and possibly also through `stdout` on the SUT.

- The complete set of test scripts is made available if the **Run Startup & Prepare for Scripts** button is pressed instead of the **Run Startup Only** button.

- Immediately after either the **Run Startup Only** or the **Run Startup & Prepare for Scripts** buttons has been pressed, A sample `impirun` command line is shown to the tester, in the scrolling output window on the Test Tool. This command line includes the specific `HOST:PORT` needed as a command-line parameter to the IMPI clients. The tester must then run the IMPI test interpreter on the SUT using this command-line, or the equivalent command-line if the IUT uses a different syntax.

  In the future, a command-line template may be made available so that the tester can specify the required syntax for the IUT, with place holders for varying parameters such as the number

[1]This connection will be disconnected after a predetermined timeout period of inactivity.

of processes. This will allow the Test Tool to display the command-lines in the correct syntax for the IUT. This should help avoid errors in starting the IUT correctly for the various tests to be performed. Of course until then, the tester can write a script to parse the sample command line and call their own `mpiexec` or `mpirun` routine properly.

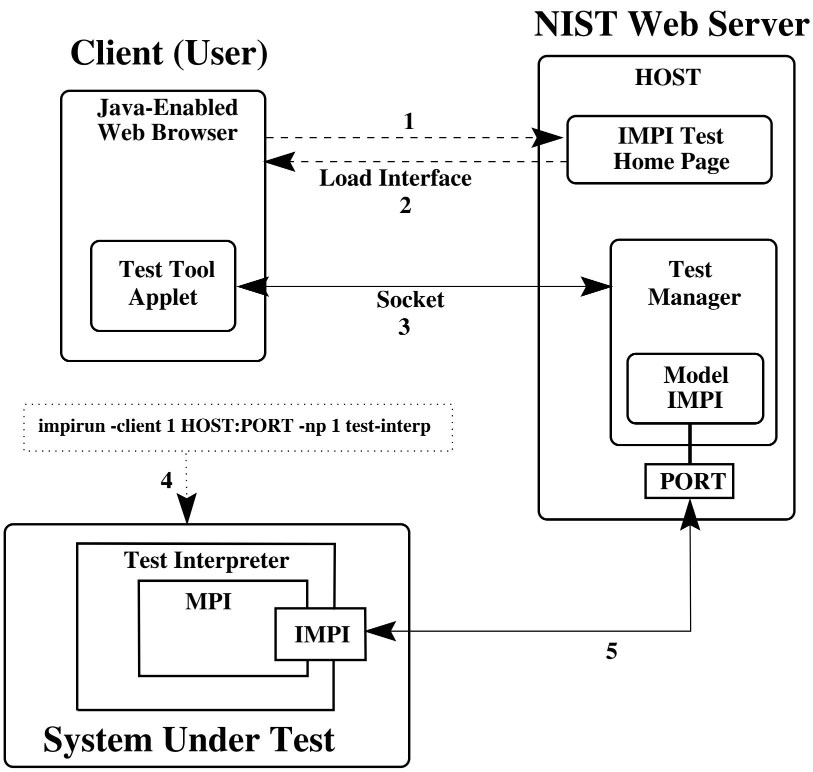

Figure 5.1: IMPI Test Architecture

Figure 5.3 shows the levels of communications protocols involved in this testing. We are most interested in verifying the operation of the IMPI level of this communications stack so the tests originate in the MPI level (an *upper*-tester). We also have a *lower*-tester that is able to examine the IMPI protocol packets as they are received from the SUT. This lower-tester has been implemented by instrumenting the *Model* IMPI *implementation* directly. The Model IMPI implementation is part of the IMPI Test Manager.

Once the tester starts the IMPI test interpreter on the SUT, the IMPI startup protocol begins between the IUT and the Model IMPI implementation running on the NIST IMPI test server. If the startup protocol succeeds, the IMPI Test Manager will present to the tester a menu of options. The format used to display this menu will likely change as the number of available tests increases. If the startup protocol fails, error recovery will be attempted and as much information as possible about the startup negotiations will be provided.

Assuming the startup protocol succeeded, the tester may select a test or group of tests to be run. The Test Manager will then begin running the tests by sending each test, one at a time, to the processes running on the SUT. Each test will determine the result, either Pass/Fail/Indeterminate, and this result will be sent to the Test Tool for display (see Figure 5.4). Once all of the requested tests have been run, the tester may select more tests or discontinue testing.

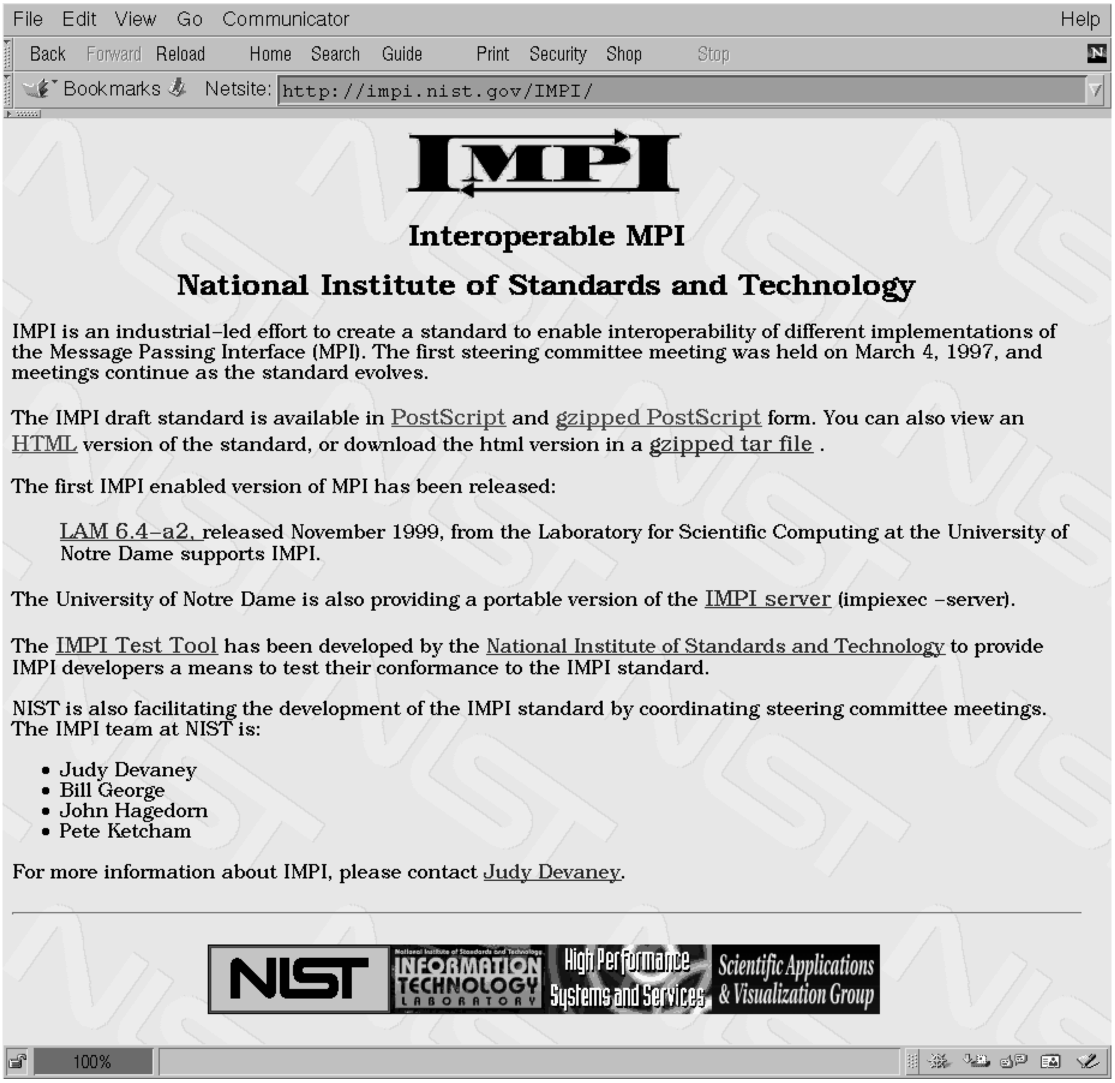

Figure 5.2: IMPI Home Page

For each test, the tester may obtain, through the IMPI web page, the source for the script that is being interpreted. We might eventually also provide hyper-links into the IMPI or MPI documents for the text that specifies the part or parts of the protocol that this code is intended to test. To keep a permanent record of the results of the tests run, the *Results* window in the Test Tool (see Figure 5.4) can be mailed to the tester, or the contents of this window can be cut directly from the window and pasted elsewhere.

If you are running many tests, it helps performance to periodically clear the *Results* window. The buffering of unlimited amounts of output tends to slow down the Test Tool.

The full testing of the IUT must include varying the `impirun` command-line options. The command-line can specify the relative ranks of the SUT nodes within the `MPI_COMM_WORLD` group. Also, tests must be made using a single node of the SUT as well as multiple nodes. The tester must therefore follow instructions given in the Test Tool as to the proper `impirun` command-line to execute each time the test interpreter is started. Although it would be preferable to automate this,

we do not yet, and probably will never, have this capability. However, we will at least provide the tester with command-lines in the Test Tool window to cut & paste.

Starting with Java SDK version 1.1, it will be possible to gain access to the disks on the machine running the Test Tool, as well as run external programs on that machine. This will be allowed if the tester certifies that the Test Manager is trusted. How this will be implemented in the browsers is not yet known. Many Web browsers in use are still using Java SDK 1.0, and those that have been updated to version 1.1 have not implemented this feature, so this is not yet an option. Even with this ability, it is not clear that we will be able to automate the running of `impirun` on the SUT.

Tests will also be provided to allow additional IMPI/MPI implementations to participate in the tests, in addition to the IUT. These tests will be needed to fully exercise the IMPI/MPI protocols.

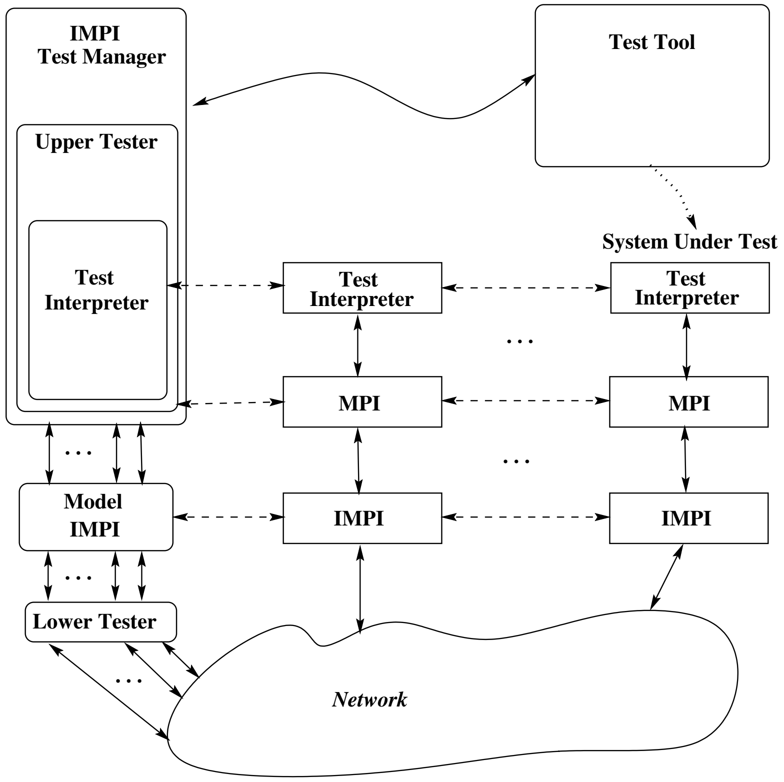

Figure 5.3: IMPI Test Communications Stack

5.2 Test Tool Applet

Figure 5.4 shows a sample of the current interface. The Test Tool is shown here after running 2 collective communications tests, one for `MPI_Barrier()` and one for `MPI_Bcast()`. The green (or gray if you do not have a color copy of this document) highlighting of the test names indicate that the tests have passed. Tests that fail would be highlighted in red and tests that have indeterminate results would be highlighted in yellow. The most up-to-date instructions on using the tester can always be found on the IMPI web site at `http://impi.nist.gov/IMPI/ImpiTIAIntro.html`.

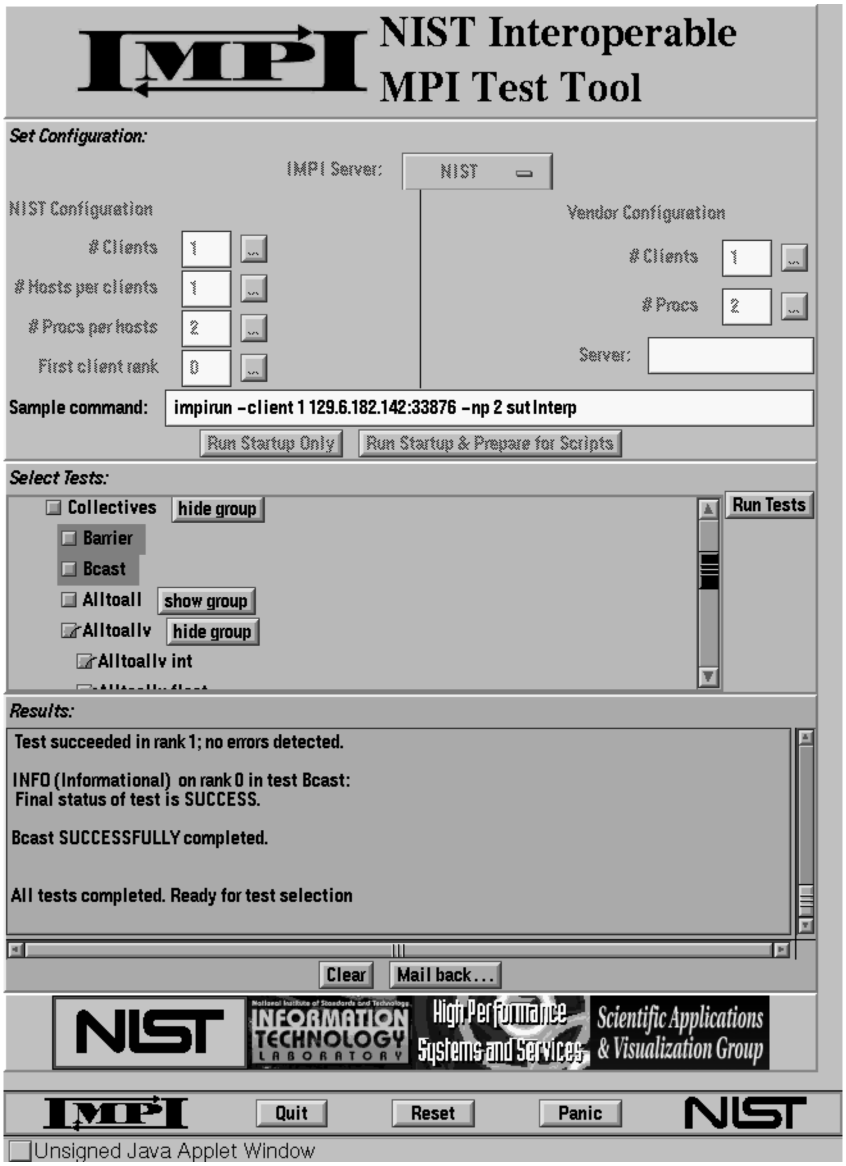

Figure 5.4: The Test Tool

5.3 Test Interpreter

Tests are written as scripts that are interpreted within a standard C MPI program on the system under test. These test scripts are delivered to the MPI processes as arrays of characters via `MPI_Send()`. The first test to be run each time the interpreter is started is a test that is embedded in the interpreter. This initial test verifies that this delivery mechanism is working. If this initial test fails, no other tests are attempted and diagnostic messages are sent to the Test Tool. For the special case of the **Startup Only** testing, this initial test of communications is not attempted.

Once the basic delivery of test scripts has been verified, the tester may select tests to be run. At this point, in the event of a test failure, it is most important to help testers diagnose the error. For this reason, the contents of each script will be available on request via the Test Tool. This section describes the operation of the interpreter and the language that it accepts so that the tester can read these scripts and hopefully diagnose the failure. Error messages produced by these scripts will also be more understandable given the test script that produced them.

The test interpreter is a C/MPI program that runs on the SUT. This program runs a loop that repeatedly receives and interprets small scripts that superficially look like standard C and MPI code. The interpreter repeats this loop until a script calls a special function which signals the interpreter to exit. The language that the interpreter understands is limited to a small subset of C with MPI function calls available. The MPI routines in the interpreter are wrappers to the actual MPI library routines in the IUT. Declarations for variables and single dimensioned arrays of the basic data types are allowed as well as control structures such as `if`, `while`, and `for`. In addition to the MPI routines, other routines are available such as `printf()` for printing to `stdout` and `report()` for passing information to the Test Manager. Details of the language accepted by this interpreter are available in the source code. A test is comprised of a block of statements in this language. Some sample tests are shown below.

The same interpreter, with some modifications, is executed within one or more *simulated* MPI processes which are running as part of the Test Manager. These simulated MPI processes execute the same script as the IUT. The MPI routines in this case are linked to routines, internal to the Test Manager, which implement the communications between the simulated processes as well as external MPI processes. Other modifications to the interpreter allow these simulated MPI processes to interact directly with the Test Manager, supplying test status and error information as the script is interpreted.

Upon completion of each test script, a short handshake protocol is executed. This synchronizes the processes between tests, although it is not a complete barrier. As part of this handshake, each process informs the *master process* of the level (integer) of the most severe error encountered during the execution of the current test script. In this context, the *master process* is the process with the lowest `MPI_COMM_WORLD` rank on the NIST side, and has no relation to the IMPI defined master processes. In this *end-of-test* handshake, the *master process* sends a ”DONE” message to every rank. They recv and verify this and send back ”done”. The master rank receives and verifies each ”done” message. See the `end_of_test_handshake()` routine in `sutInterp.c` for more details. If a test script appears to have completed but the tester is hung, the processes may be stuck in this final handshaking protocol indicating that one or more processes have not made it to the end of the current test script.

To distinguish between the completion of the current test script and the completion of the final handshake, observe the following printouts:
At the end of a test script, the message:
`END EXECUTING rank` *r*

will be printed, where r is the process rank. This is printed *before* the final handshake. After the final handshake, the message:

. . . . *script name* `rank` r `done` . . . .

will be printed.

Here is a simple test that exercises `MPI_Send`. Some of the comments refer to the test while other comments refer to the syntax of the interpreted language.

```
{
  int i;  /* Only one declaration allowed per line */
  int in;
  int my_rank;
  int nprocs;
  int status[3];
  int nerrors;

  /* Note:  & not used in interpreter (no pointers). */
  MPI_Comm_rank(MPI_COMM_WORLD, my_rank);
  MPI_Comm_size(MPI_COMM_WORLD, nprocs);

  if (my_rank > 0) {

    /* All but node 0 sends their rank to node 0. */
    MPI_Send(my_rank, 1, MPI_INT, 0, my_rank, MPI_COMM_WORLD);

  } else {

    nerrors = 0;
    for (i=1; i<nprocs; i++) {
      MPI_Recv(in, 1, MPI_INT, MPI_ANY_SOURCE, MPI_ANY_TAG,
               MPI_COMM_WORLD, status);
      if (status[MPI_TAG] != status[MPI_SOURCE]) {
        nerrors = nerrors + 1; /* ++ and -- are not available */

        /* report() acts like a printf statement when executed
         * on the IUT.  Otherwise, report() sends this info to the
         * Test Manager, which will pass this info on to the
         * Test Tool. All numeric values are printed using the
         * %f format.
         */
        report("Error: source:%.0f != Tag:%.0f",
               status[MPI_SOURCE], status[MPI_TAG]);

      } else if (in != status[MPI_TAG]) {
        nerrors = nerrors + 1;
        report("Error: received:%.0f != Source,Tag:%.0f",
               in, status[MPI_TAG]);
      }
    }
    if (nerrors == 0) {
      report("Result: Pass");
    } else {
      report("Result: Fail: %.0f error out of %.0f messages",
```

```
                  nerrors, nprocs-1);
        }
      }
    } /* end of script */
```

For the preceding test to pass, MPI process rank 0 must receive a message from each other process with the tag and the single integer both matching the source process rank. This test will usually be run with rank 0 owned by the Test Manager, although this is not required. Tests may have requirements as to the number of processes and the ordering of them in `MPI_COMM_WORLD`. These restrictions must be enforced by the script itself when the tests are executed; the Test Manager has no specific information about these scripts to allow it to allow or deny any particular test.

```
    {
      int i;
      int p;
      int a[5];
      int answer;
      int correct_answer;
      int root;
      int rank;
      int nprocs;
      int scale;

      root = 0;
      MPI_Comm_rank(MPI_COMM_WORLD, rank);
      MPI_Comm_size(MPI_COMM_WORLD, nprocs);

      if (nprocs > 5) {
        report("Test skipped. Too many processes");
        return;
      }

      scale = 1;
      for (i=0;i<rank;i=i+1) {
        scale = scale * 10;
      }

      for (i=0;i<5;i=i+1) {
        a[i] = scale;
      }
      MPI_Reduce(a, answer, 10, MPI_INT, MPI_SUM, root, MPI_COMM_WORLD);
      if (rank == root) {
        /* Calculate the correct answer */
        scale = 1;
        correct_answer = 0;
        for (p=0;p<nprocs;p=p+1) {
          correct_answer = correct_answer + 5 * scale;
          scale = scale * 10;
        }
        if (answer != correct_answer) {
          report("Result: Fail, expected %.0f != %.0f",
```

```
                    correct_answer, answer);
          } else {
            report("Result: Pass");
          }
        }
      }
```

Each node in the preceding test fills an array and then calls the MPI_Reduce() routine. The correct answer is computed, based on the number of nodes involved in the test, and the root node verifies that the correct value has been received. The Test Manager's simulated MPI processes actively participate in the algorithms used to perform this, and other, collective operations.

The interpreter must be able to execute test scripts that exercise all aspects of the IMPI protocols. Many test scripts have been written and more will be added as needed. The first tests implemented focussed on the IMPI start-up protocol and the basic MPI routines, MPI_Init(), MPI_Finalize(), MPI_Send(), and MPI_Recv(). As of this writing, this tester can also exercise MPI_Sendrecv(), the non-blocking point-to-point routines MPI_Isend() and MPI_-Irecv(), MPI_Iprobe(), all of the collective communications routines, as well as the communicator handling routines MPI_Comm_create(), MPI_Comm_dup(), MPI_Comm_split(), MPI_Intercomm_create(), and MPI_Comm_Intercomm_merge(). The are over 100 test scripts currently available to exercise these routines. All scripts are available online at the NIST IMPI web site.

5.4 Test Manager

Note that the Test Manager itself knows very little about the test scripts and what they are designed to test. All of this information is implicit in the test scripts. These scripts are interpreted on all of the MPI processes including the Test Manager's simulated MPI processes.

On the NIST Web server, which runs the Test Manager, each test script is stored as a single ASCII file. During testing, these scripts are read from the disk and sent to each of the MPI processes as input to the interpreter. The Test Manager maintains a queue of requested tests and, in order to meet the configuration requirements of the requested tests, may prompt the tester as needed to restart the interpreter on the SUT. The interpreter must be restarted each time a set of tests is to be run which requires a different configuration of processes than the current configuration.

The Test Manager's version of the test script interpreter includes support subroutines, like report(), which accepts messages (text strings) from the simulated MPI processes and sends these messages on to the Test Tool applet for display, so that the tester can monitor the progress of the tests.

Bibliography

[1] Message Passing Interface Forum. MPI-2: A Message-Passing Interface standard. *The International Journal of Supercomputer Applicatons and High Performance Computing*, 12(1-2), 1998.
